# Malaria mosquito antimicrobial defence requires immunity and detoxification gene regulation by Lola

**DOI:** 10.1111/imb.70051

**Published:** 2026-06-20

**Authors:** Heidi Espadas‐Álvarez, Verónica Valverde‐Garduño

**Affiliations:** ^1^ Laboratorio de Genética Molecular, Centro de Investigaciones Sobre Enfermedades Infecciosas Instituto Nacional de Salud Pública Cuernavaca Morelos Mexico; ^2^ Facultad de Ciencias Universidad Nacional Autónoma de México, Ciudad Universitaria Ciudad de México Mexico

**Keywords:** anopheles, antimicrobial defence, detoxification gene regulation, immunity gene regulation, malaria mosquito

## Abstract

Malaria‐causing *Plasmodium* parasites are transmitted by some *Anopheles* mosquito species. The genetic and immunity basis for vector competence, the mosquitoes' intrinsic ability to transmit pathogens, has been extensively studied in major vectors such as *Anopheles gambiae* and *Anopeles stephensi*. In *An*. *gambiae*, Lola‐type motifs were identified in immunity‐related *cis*‐regulatory elements; the Lola transcription factor is known to be involved in *Drosophila* development. Here, the potential role of *lola* in mosquito antimicrobial defence was investigated in *Anopheles albimanus*, a malaria vector in the Americas. The in vivo attenuation of *lola* was found to compromise mosquito survival to microbial infection. It was also found that the induction of antimicrobial defence genes requires *lola* upregulation. Genes for antimicrobial peptides, C‐lectins, pattern recognition receptors, Clip‐domain serine proteases and ML‐domain proteins showed *lola* upregulation dependency. Enzymes involved in the generation and detoxification of reactive oxygen species, as well as components of the JNK immune signalling pathways, are also dependent on *lola* upregulation. These findings suggest that *lola* regulates the expression of immunity and detoxification genes and that there is a functional link between JNK signalling and *lola* expression in the mosquito midgut. Dysregulation of this pathway may contribute to increased pathogen invasion and malaria transmission.

## Graphical abstract

- Malaria mosquito antimicrobial defence requires upregulation of *lola*. Attenuation of *lola* in the midgut of *Anopheles albimanus* mosquitoes inhibits the upregulation of immunity genes induced by challenge.
- Putative target genes of the Lola transcription factor were revealed by *lola* attenuation, including Cecropin‐C, Draper, PPAF2, Clip‐domain serine proteases, JNK, Kayak and other antimicrobial defence genes.
- Lola activity in upregulating immunity and other antimicrobial defence genes is required for adult mosquito survival during microbial challenge. Attenuation of *lola* compromises survival upon exposure to live bacteria.

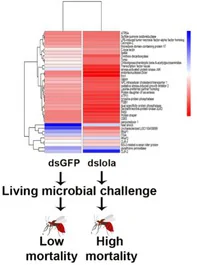

## INTRODUCTION

Some *Anopheles* mosquito species are among the deadliest insects because they are vectors of *Plasmodium* parasites that cause malaria. The World Health Organization (WHO) reported 253 million cases of malaria and 597,000 deaths in 2023 (WHO, 2024). Some of them also transmit viruses that cause human diseases (Terradas et al., 2023). Vector competence is the intrinsic ability of mosquitoes to transmit pathogens and is directly related to their genetics and immunity (Hardy et al., 1983). Mosquitoes lack acquired immunity, but their innate immune system can destroy various pathogens (Wang et al., 2013). Immunity plays an important role in the interaction between a pathogen and its vector host. Studies have shown that innate immunity plays a key role in pathogen‐vector interactions and that pathogens encounter multiple barriers within the mosquito's innate immune system. Four pathways control the mosquito immune response: Toll, immune deficiency (Imd), Janus kinase signal transducer and activator of transcription (JAK–STAT) and mitogen‐activated protein kinases (MAPKs) (Clayton et al., 2014; Zhang et al., 2021). In addition to regulating the innate immune response, c‐Jun N‐terminal kinase (JNK) signalling also plays an important role in the cellular stress responses, mediates antiplasmodial immunity and participates in the defence against bacteria and viruses in the fruit fly and mosquitoes (Dong et al., 2002; Silverman et al., 2003; Tikhe & Dimopoulos, 2021; Zhang et al., 2021). Very little is known about the genes regulated by JNK in mosquitoes.

Since innate immunity effectors are directly encoded in insect genomes, the ultimate regulation of effector innate immunity genes occurs mainly through transcriptional control. Accordingly, immunity transcription factors have been found to play a key role in regulating the innate immune response in insects. In mosquitoes, Rel1, Rel2, STAT‐A and STAT‐B transcription factors are nodes activated by immune pathways that upregulate innate immunity genes by directly binding to *cis*‐regulatory sequences that control gene transcription (Barillas‐Mury et al., 1999; Ehret et al., 2001; Meredith et al., 2006; Shin et al., 2005).

The JAK–STAT pathway is central to mosquito midgut innate immune response against *Plasmodium* (Bahia et al., 2011; Gupta et al., 2009). The IMD pathway responses are controlled by the transcription factor Rel2 that is activated after infection with Gram‐negative bacteria, Gram‐positive bacteria and parasites (Gendrin et al., 2017; Khan et al., 2016), the Toll pathway is regulated by transcription factor Rel1 and tends to be induced against Gram‐positive bacteria, fungi, parasites and viruses (Barletta et al., 2022; Tikhe & Dimopoulos, 2021). The search for other transcription factors that may regulate innate immunity gene expression will contribute to a better understanding of the molecular mechanisms underlying mosquito vector competence.

Previously, the genome‐wide identification of *cis*‐regulatory sequences in *Anopheles gambiae* suggested the potential participation of Lola TF in the regulation of gene expression in mosquito innate immune response (Perez‐Zamorano et al., 2017). However, it remains unknown whether any genes involved in the mosquito's antimicrobial defence are regulated by Lola. *Longitudinals lacking* (*lola*) was first identified in *Drosophila* as regulating the axon growth and guidance in the Central Nervous System (CNS) (Bass et al., 2007; Crowner et al., 2002; Giniger et al., 1994). Lola belongs to a family of transcription factors that contain the Broad‐complex, Tramtrack, Bric‐à‐brac (BTB) domain and zinc finger motif and is involved in a wide variety of developmental pathways (Ohsako et al., 2003; Silva et al., 2016; Zhang et al., 2003). BTB proteins function in a variety of cellular contexts, including transcriptional regulation. Alternative splicing of the *Drosophila lola* primary transcript encodes at least 25 protein isoforms; some of these isoforms are conserved in *Anopheles gambiae*, suggesting that the structural domains are likely critical to Lola function (Goeke et al., 2003). It has been suggested that the alternative forms of *lola* are responsible for different functions of this gene. Lola was recently shown to regulate adult midgut homeostasis (Zhao et al., 2022), and a microarray‐based study in the fruit fly revealed that some Lola‐dependent genes are involved in various processes, including oxidative stress (Gates et al., 2011). Although little is known to date about the role of Lola regulation in the insect innate immune response, the p38Kb genomic locus has been found to contain Lola consensus‐binding sites. The overexpression of Lola in *D*. *melanogaster* is sufficient to induce p38Kb transcription and enhance resistance to oxidative stress (Ryan et al., 2020). In addition, a reporter assay showed that Lola silencing decreased the activity of the drosomycin and attacin promoters in *Drosophila* S2 cells (Kleino et al., 2005).

The mosquito midgut constitutes an important barrier to parasite infection, primarily due to the mosquito's immune responses (Abraham & Jacobs‐Lorena, 2004). The presence of pathogens in the midgut leads to expression of immunity genes that mediate antiparasitic and antimicrobial defences (Michel & Kafatos, 2005). Recognition of pathogen‐associated molecular patterns (PAMPs) by insect receptors is necessary to initiate immune signalling cascades that culminate in the transcription of effector genes encoding antimicrobial peptides (Clayton et al., 2014).

The majority of studies contributing to our current knowledge of malaria mosquitoes have focused on *Anopheles gambiae*, *Anopheles coluzzi* and *Anopheles stephensi* because of the high mortality and morbidity they cause. *Anopheles albimanus* is a major vector of malaria parasites in the Americas (Sinka et al., 2010), but it has been studied much less than the above‐mentioned species. The present study aimed to investigate whether Lola may play a role in regulating the expression of antimicrobial defence genes in the midgut of the mosquito *An. albimanu*s.

Here, a reverse genetics approach was combined with differential gene expression analysis using RNA‐seq to investigate whether Lola may participate in mosquito antimicrobial defence. Double‐stranded RNAs encoding part of the *lola* gene (dslola) and part of GFP (dsGFP), respectively, were synthesized by in vitro transcription. Using these molecules in vivo, several Lola target genes involved in different pathways of the innate immune response were identified. Our results provide further insight into the regulation of immunity gene expression of one of the major vectors of human malaria. These results provide evidence that Lola's function is conserved between *Drosophila* and *Anopheles*, as previously suggested in Perez‐Zamorano et al. (2017).

## MATERIALS AND METHODS

### Mosquito rearing and maintenance

Experiments were performed using female mosquitoes of the *An. albimanus* Tapachula strain. The colony was maintained at the insectary at the National Institute of Public Health (Instituto Nacional de Salud Pública, Cuernavaca, México). Adults were maintained under standard rearing conditions (RH 70% ± 10%, 27 ± 3°C, 12 L: 12D photoperiod). Blood‐fed adult females were allowed to lay eggs. On the day of hatching, larvae were seeded into plastic trays (21 cm × 15 cm × 10 cm) containing 1 L of dechlorinated water at a density of 200 individuals per tray. Larvae were provided with 200 mg of ground cat kibble 2 days after hatching. Upon pupation, trays were placed inside an emergence cage (19 cm high × 16 cm diameter) and provided with a diet of 10% sucrose ad libitum for emerging adults. Tissue dissection was performed on adult females aged 3–5 days.

### Live bacteria challenge

Adult females were injected with dsRNA 3–5 days postemergence, and 1 day later, they were injected with *Escherichia coli*, a Gram‐negative bacterium.

Glycerol stocks of bacteria were used to inoculate LB broth cultures, which were grown in a shaker overnight at 37°C. An Implen NanoPhotometer NP80 was used to measure optical density (OD600). Bacterial cultures were grown in LB broth, 1 mL culture (approximately 3 × 10^6^ bacteria) were centrifuged for 5 min. The supernatant was discarded; the pellet was washed twice with PBS and resuspended in 200 μL of PBS. Mosquito survival was monitored daily for 16 days after intrathoracic injection of 0.5 μL of bacterial suspension. Mosquitoes that died within 24 h of injection were not considered in the analysis. Approximately 25 mosquitoes were used for each group of injected mosquitoes, and three replicates were performed for all experiments. Survival patterns were determined using Kaplan–Meier survival analysis, and a log‐rank test was used to assess significance in GraphPad Prism.

### Heat‐killed bacteria challenge

For bacterial challenge, heat‐killed *E. coli* (ATCC29922) and *Micrococcus luteus* were suspended at 5 × 10^7^ bacteria/μL. Adult female mosquitoes were injected with 0.5 μL of a 1:1 bacterial mix and anaesthetised on ice for 5 min. The tissue was collected 3 h after microbial challenge.

### Tissue collection

For expression profiles, abdomens (containing the fat body), head, midgut regions (proventriculus, anterior and posterior midgut), total midgut, Malpighian tubules and ovaries were dissected. The dissected midgut regions were pooled (100 for proventriculus and anterior midgut). Tissues were maintained in ice‐cold Schneider's insect culture medium (Gibco) to avoid degradation, and fresh tissues were immediately processed for RNA analyses. For RT‐qPCR experiments, a minimum of 5 individuals were dissected per replicate; for silencing experiments, 10 individuals were dissected. For all experiments, a minimum of two replicates was prepared. Haemocytes were pooled from at least 10 adult mosquitoes for each experimental RNA isolation set, following the methods of Qayum and Telang (2011). After removal and pooling, they were stored at −80°C until RNA extraction.

### Gene knockdown assays in vivo

For double‐stranded RNA (dsRNA) preparation: A 403 bp fragment of gene *lola* AALB004332 (genomic coordinates: AalBS2_3L:7,423,169‐7,457,333(‐) VectorBase (Giraldo‐Calderon et al., 2015) was amplified by PCR using primers to amplify portions of one specific exon, genomic coordinates of the primer binding locations was AalBS2_3L: 7,423,183‐7,423,200(‐) forward primer and AalBS2_3L: 7,423,568‐7,423,585(‐) reverse primer. The dsRNAs were synthesized using the T7 RiboMAX™ Express RNAi System (Promega) according to the manufacturer's instructions. For control, green fluorescent protein (GFP) dsRNA was produced from a 400 bp region based on sequences derived from *Aequorea victoria* (GenBank: MN114103.1), with genomic coordinates 82 to 474.

### RNA isolation, cDNA synthesis and quantitative PCR (qPCR)

To analyse the expression profiles of *An. Albimanus‐lola* and their expression profiles after bacterial challenge, total RNA was extracted from mosquitoes using TRIzol reagent (Invitrogen) according to the manufacturer's protocol. Samples were then extracted by a standard phenol‐chloroform procedure. Total RNA quantity was measured with a Nanodrop spectrophotometer. For cDNA synthesis, 1 μg of DNase‐treated RNA was used with the GoScript reverse transcription supermix (Promega). cDNA was diluted 1/10 in RNase/DNase‐free water before using it as a template in qRT‐PCR experiments. In each 10 μL qRT‐PCR reaction, one μL cDNA was used. qRT‐PCR was performed on a CFX Touch Real‐Time PCR Detection System (Bio‐Rad), using SYBR Green master Mix (Takara) and *lola‐*specific primers (Table S2). Primers were also designed for a ribosomal protein subunit S7 gene, which was used as a housekeeping control. Melting curves were obtained for each data point to validate the specificity of the PCR reactions (Figure S3). All reactions were performed with 30 s at 94°C, followed by 40 cycles of 30 s at 94°C, 30 s at 58°C (60°C for S7) and 30 s at 72°C, and a melt curve was analysed at 65–95°C. Relative expression was calculated using the 2^−∆∆Ct^ method. Experiments were replicated with two different mosquito pools.

### RNA sequencing

Mosquitoes treated with *lola* dsRNA and GFP dsRNA were collected 24 h post‐injection for RNA sequencing. Twenty‐five midguts were pooled for each sample, and three biological replicates were used for each treatment. Total RNA was extracted using TRIzol® reagent according to the manufacturer's instructions (Invitrogen). All samples RNA‐seq were homogenized in 750 μL of TRIzol reagent and stored at −80°C. RNA was extracted via the chloroform method. In brief, TRIzol samples were thawed, mixed with 200 μL of chloroform, vortexed and centrifuged for 5 min at 14,000 rpm at 4°C. The aqueous supernatant was recovered from each tube, and RNA was precipitated overnight with isopropanol at −80°C. The pellet was recovered in 800 μL of 100% ethanol and frozen at −80°C. Libraries were pooled and sequenced on the Illumina NextSeq 500.

### RNA‐seq differential expression analysis

The raw sequences (FASTQ) were subjected to a quality check using FastQC (http://www.bioinformatics.babraham.ac.uk/projects/fastqc/). Adapter removal and quality trimming of the raw reads were performed using Trimmomatic v0.39 (Bolger et al., 2014). The trimmed paired reads were mapped to the *An. albimanus* STECLA reference genome, AalbS3 (Compton et al., 2020), using STAR v.2.7.5 (Dobin et al., 2013). We used FeatureCounts (Liao et al., 2014) to quantify transcript abundance in each sample using the gene annotation *An. Albimanus* STECLA strain AlbS3 of VectorBase (Giraldo‐Calderon et al., 2015). The DESeq2 package performed differential expression (DE) analysis between sample groups, was used to calculate mean variance and obtain differentially abundant transcripts between control (basal condition) and treatment (challenge condition) replicates using default parameters, Wald's hypothesis tests according to the criteria of adj.P. Val < 0.05 and logFC > 1. Two comparisons were analysed: silenced versus non‐silenced groups. All principal component analyses (PCA), heatmaps, volcano plot visualizations and plotting were performed in R. Venn diagrams were generated using jvenn.

### Gene ontology and pathway annotation

Differentially expressed genes were annotated by importing gene names and descriptions from VectorBase. Processes, functional groups and pathway analyses of DE genes were annotated using the Kyoto Encyclopedia of Genes and Genomes (KEGG) (Kanehisa & Goto, 2000). Pathway painting was performed using KEGG and SRplot (Tang et al., 2023). Gene Ontology (GO) analyses were conducted using ShinyGO (http://bioinformatics.sdstate.edu/go/) (Ge et al., 2020).

### Validation of RNA‐seq differential expression by real‐time quantitative PCR

A total of 5 genes identified by RNA‐seq as differentially expressed between dsGFP (basal and challenge) and dslola (basal and challenge) mosquitoes were selected for real‐time quantitative PCR analysis. Post‐silencing and challenge, total RNA was extracted from midgut samples using TRIzol® reagent according to the manufacturer's instructions (Invitrogen). First‐strand cDNA was synthesized using Maxima H Minus cDNA Synthesis Master Mix with dsDNase (Thermo Fisher Scientific). PCR reactions were run on a StepOnePlus (Applied Biosystems). No primer‐dimer was detected in the melting‐curve analysis, and primer pairs with amplification efficiencies greater than 90% were selected (Figure S3). Gene expression was quantified by qPCR using SYBR Green master Mix (Takara). Ribosomal protein subunit S7 gene expression was used for normalization. Reactions were performed with the following cycle conditions: an initial denaturation at 95°C for 30 s, followed by 40 cycles of 95°C for 30 s and 60°C for 30 s; a final extension at 72°C for 30 s, and a melting‐curve analysis. The ∆∆Ct method was used to calculate relative fold changes, as described previously (Schmittgen & Livak, 2008).

### Hydrogen peroxide assay in the midgut

Hydrogen peroxide was measured using Amplex Red (Invitrogen) according to the manufacturer's recommendations. Five adult female midguts per group were dissected and collected in 100 μL of Ringer's solution (pH 7.3), 24 and 72 h after silence. Dissected midguts were homogenized and centrifuged to remove debris. Hydrogen peroxide level in the adult midguts was monitored by the addition of 50‐μL reaction buffer (100 μM Amplex Red, Invitrogen # A12222; 0.2‐U/mL horseradish peroxidase prepared in Reaction Buffer) to 20 μL homogenized midgut extract. After a 30 min incubation, the epithelia were evaluated for absorbance emission at 530/560 nm (Ex/Em) in an Implen NanoPhotometer. A standard curve was plotted with a range of hydrogen peroxide dilutions (0–10 μM) and used to determine the amount of hydrogen peroxide.

### Quantification of endogenous mosquito midgut bacteria

Quantification of bacterial load was performed using qRT‐PCR as described above. To quantify the microbiota relative load, the 16S rRNA gene was amplified from mosquitoes 2 days after injection with dslola or dsGFP (control). All qPCR measurements were performed with two technical replicates. Differential gene expression was evaluated using the 2^−∆∆Ct^ method and statistically analysed using Student's *t‐*test. The sequences of universal 16S primers (Sharma et al., 2020) are listed in Table S2.

### Statistical analysis

Data were analysed using GraphPad Prism 7 software. Statistical significance between two experimental groups or among multiple experimental groups was evaluated using Student's *t*‐test and one‐way analysis of variance with Tukey's post hoc test, respectively. A value of *p* < 0.05 was considered significant.

## RESULTS AND DISCUSSION

### Expression profile of *lola* in *An. albimanus* tissues

**FIGURE 1:**
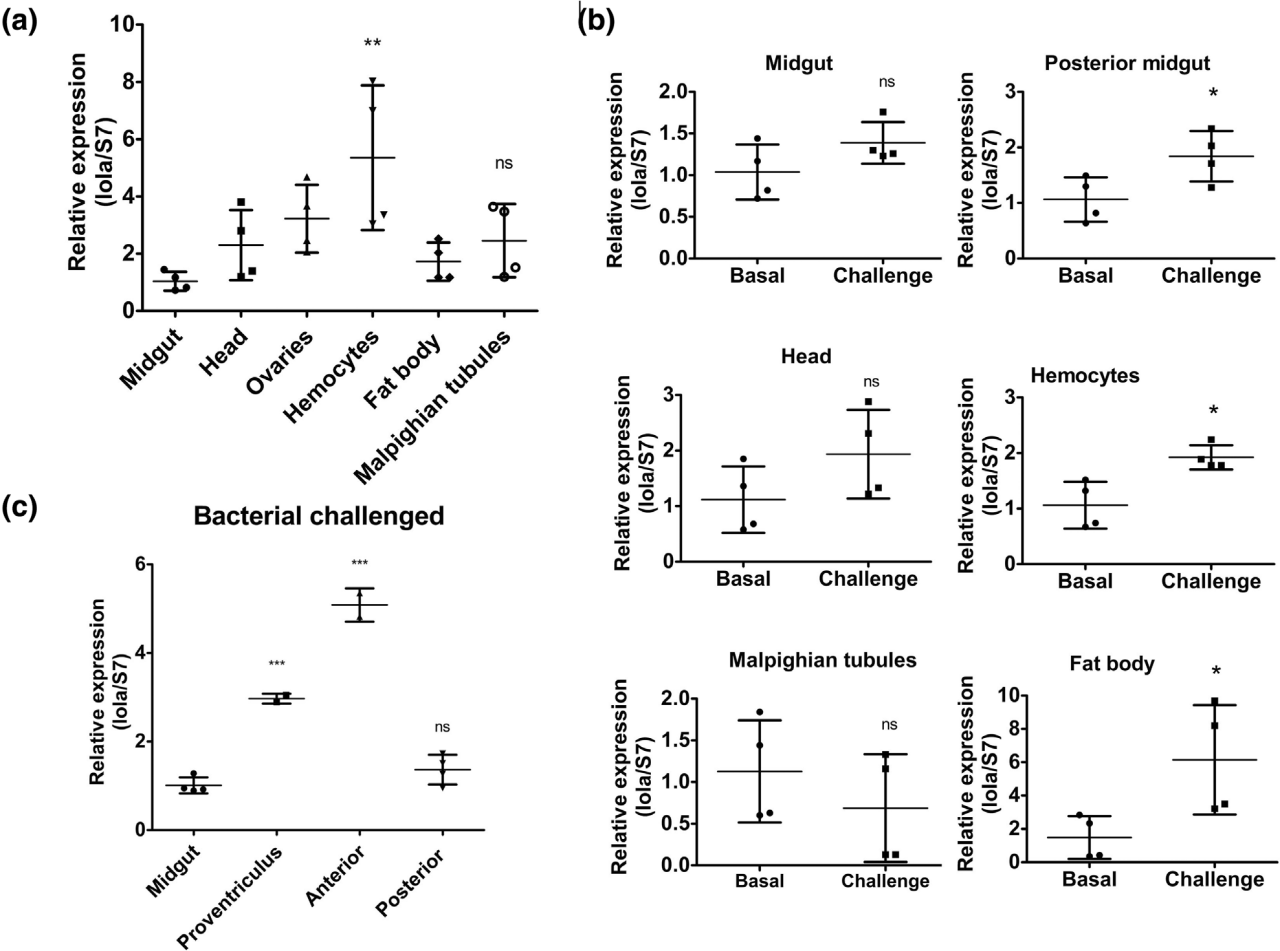
Tissue expression pattern of *lola* in *Anopheles albimanus*. (a) Relative gene expression of *lola* in the head, total midgut, ovary, fat body, Malpighian tubules and hemocytes of 3–5‐day‐old sugar‐fed females, detected by quantitative reverse transcription PCR (qRT‐PCR). The mRNA levels in the midgut were considered the control, set to 1.0. Asterisks indicate significant differences. Error bars indicate mean ± standard deviation (SD). Results of two independent experiments are shown (**p < 0.05), One‐way ANOVA and Tukey's post hoc test. (b) Comparative levels of the *lola* gene were analysed after 3 h of bacterial challenge in tissues, cells and the midgut. The relative fold induction was calculated relative to sugar‐fed mosquitoes. (c) Comparative levels of the *lola* gene after 3 h of bacterial challenge in the regions proventriculus anterior and posterior midgut. Significant differences (upregulation) in mRNA levels between control and challenged mosquito tissues are indicated by asterisks. The mRNA levels in the challenge‐midgut were considered the control, set to 1.0. Error bars indicate mean ± standard deviation (SD). Significance was determined by Student's *t*‐test, *p < 0.01. Results of two independent experiments are shown. The error bars represent the SD for three independent assays, and ns (not significant) were indicated with each set of quantification.

Here, the *An. albimanus* ortholog of *lola Drosophila* was identified by sequence alignment of the mosquito gene AALB004332 (Vectorbase), which encodes the protein A0A182FCU6 (Uniprot). The similarity to the fly transcription factor is 78% at the protein sequence level. The sequence for this gene encodes an N‐terminal region containing the BTB domain, and the 3′ variable sequences of the gene encode the C‐terminal domain containing zinc finger motifs. The pattern of tissue expression of *lola* mRNA in *An. albimanus* was unknown. Whether *lola* participates in insect immune responses is also currently unknown. To determine the pattern of expression of *lola* in *An. albimanus* tissues, we analysed the expression of this gene by qPCR in the head, ovaries, proventriculus, anterior and posterior midgut, Malpighian tubules, abdomen (containing the fat body) and hemocytes. Transcripts of *An. albimanus‐lola* were most abundant in the hemocytes (5.3‐fold), compared with the other mosquito tissues (Figure 1a). No expression was observed in the proventriculus and anterior midgut in sugar‐fed mosquitoes.

### Immunity challenge induces upregulation of *An. albimanus‐lola*

To investigate whether the transcriptional regulation of *lola* is involved in the mosquito *An. albimanus* innate immune response, we analysed *lola* gene expression after an in vivo bacterial challenge. A mixture of heat‐killed Gram‐positive bacteria and Gram‐negative bacteria was used to challenge mosquitoes. Upregulation of *lola* expression was significantly induced in three immune‐responsive mosquito tissues: the midgut, fat body and haemocytes (Figure 1b). The bacterial immune challenge‐induced upregulation of *An. albimanus‐lola* in posterior midgut (1.8‐fold), fat body (6.1‐fold) and haemocytes (1.9‐fold) (Figure 1b). Interestingly, the expression of *lola* was induced in proventriculus 2.9‐fold and anterior midgut (5.0‐fold) compared with total midgut (Figure 1c). These results show that the transcript abundance of this gene is modulated by bacterial challenge, suggesting a potential functional role in mosquito immune defences. Next, we investigated whether lola expression affects the expression of other mosquito genes.

### Expression of *An. albimanus‐lola* is attenuated by dsRNA in the midgut

Here, we used RNAi to knock down (KD) *An. albimanus‐lola* expression to determine whether the Lola transcription factor may participate in *An. albimanus* innate immune response. This type of RNAi‐based reverse genetic technique has been widely used to characterize interactions between vectors and pathogens. The RNAi method using dsRNA (Catteruccia & Levashina, 2009) was applied here to determine whether in vivo expression of this gene, which encodes a newly identified *An. albimanus* transcription factor, is required for the innate immune response. Silencing dsRNA included a segment of the *lola* coding sequence (AalBS2_3L: 7,423,169‐7,457,333(‐). The silencing efficiency of *lola* KD in the midgut was 73% at 24 h post‐injection (pi) and 92% at 72 h (pi), respectively, compared with the injection of mosquitoes with dsRNA coding for a GFP fragment of similar length (genomic coordinates 82 to 474) at the same tissue and time (Figure S1). Therefore, the expression of *lola* was efficiently downregulated in the midgut of *lola* dsRNA‐injected mosquitoes, in contrast with GFP dsRNA‐injected mosquitoes (controls). This dsRNA was then used to attenuate *lola* expression and investigate potential targets that could require *lola* to regulate the expression of immunity genes and/or genes from other mosquito physiological pathways induced in the mosquito midgut after microbial challenge.

### Attenuation of *lola* compromises survival after a living microbial challenge

**FIGURE 2:**
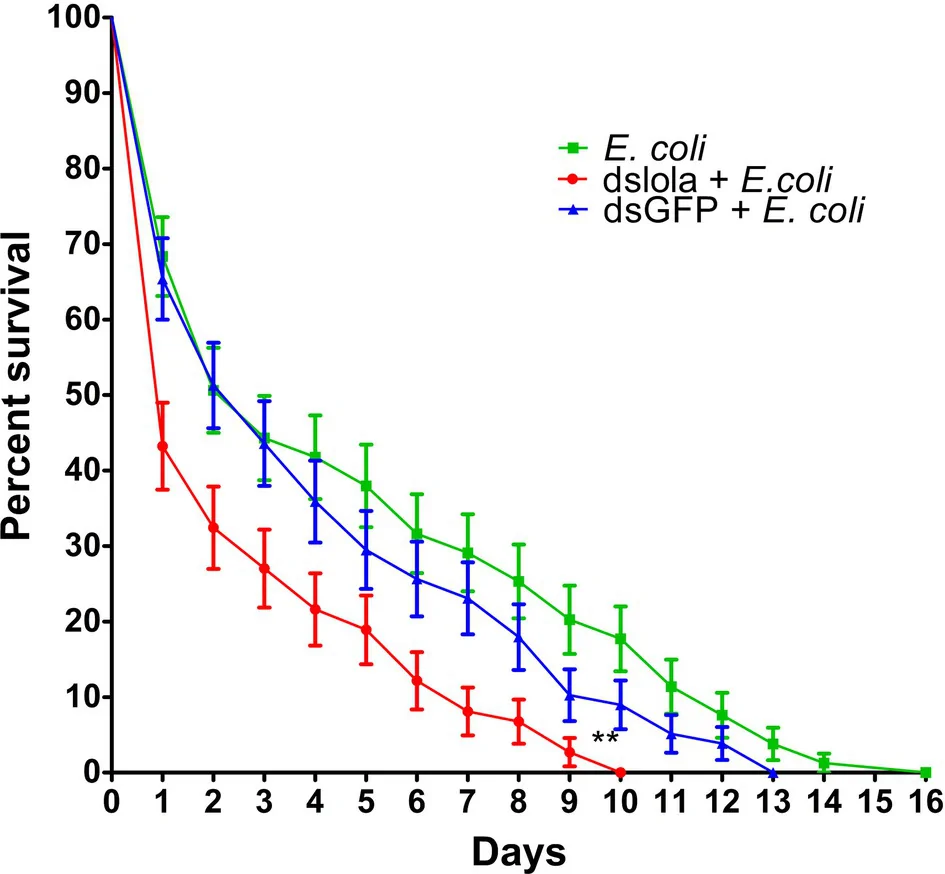
Effect of *lola* knockdown on mosquito mortality. Lola silencing with dsRNA resulted in decreased mosquito survival after challenge with *Escherichia coli* compared with the challenged control GFP dsRNA‐treated (GFP) mosquitoes. *E. coli*. (green squares), dsGFP (blue triangles) and dslola (red circles) are shown. Each experiment was performed with 25 mosquitoes, and plots include data from three independent biological replicates. The error bars represent the standard deviation. Survival curves were compared using the Log‐Rank survival test. Kaplan–Meier survival analysis was used with the log‐rank (Mantel–Cox) test for significance evaluation. Two asterisks indicate a significant difference (*p* < 0.001).

The potential involvement of Lola in adult mosquito antimicrobial defence was investigated using RNAi analysis. Attenuation of *lola* expression severely compromised adult mosquito survival upon exposure to live *E. coli*. We observed an average decrease of 3 days in mosquito survival after injection of *lola* dsRNA (+*E. coli*) compared with dsGFP (control + *E. coli*), and an average decrease of 6 days after injection of *E. coli* only. The survival rates were also analysed using the Kaplan–Meier method. These analyses showed that the survival rate of *lola*‐attenuated mosquitoes after a challenge with *E. coli* was significantly lower than that of control *GFP dsRNA*‐treated and challenged mosquitoes (*χ* ^2^ = 8.92, df = 1, *p* = 0.0028) (Figure 2). These results indicate that Lola is required for the mosquito antimicrobial defence.

### *lola* KD induces differential gene expression after immune stimulation

A genome‐wide RNA‐seq approach was applied to identify genes potentially regulated by Lola. Differential gene expression analysis of the midgut was carried out in mosquitoes with attenuated *lola* expression, via dsRNA‐*lola* injection, and in dsRNA‐GFP‐injected control mosquitoes. Both groups were then challenged with a mixture of heat‐killed bacteria. Genome‐wide transcriptional profiling was conducted using total RNA extracted from triplicate *An*. *albimanus* pooled midgut samples from treatment (silenced) and control (non‐silenced) mosquitoes. Following sequence trimming and quality‐control bioinformatics pipelines, individual datasets were aligned to the *An. albimanus* Santa Tecla strain genome assembly AalbS3 (Compton et al., 2020). Sequencing of the basal 1, basal 2 and basal 3 (silenced, basal) midgut libraries generated more than 36 million (36,706,712) Illumina 75 bp paired‐end directional reads across all samples (Table S1). The key metric in the graph is the average quality score, as shown in (Figure S2). The results obtained from mapping processed reads (33,222,810) to *An. albimanus* STECLA (AalbS3) showed a higher percentage of uniquely mapped reads using the Spliced Transcripts Alignment to a Reference (STAR) (Dobin et al., 2013). Similar numbers of reads were obtained in the other groups (Table S1).

**FIGURE 3:**
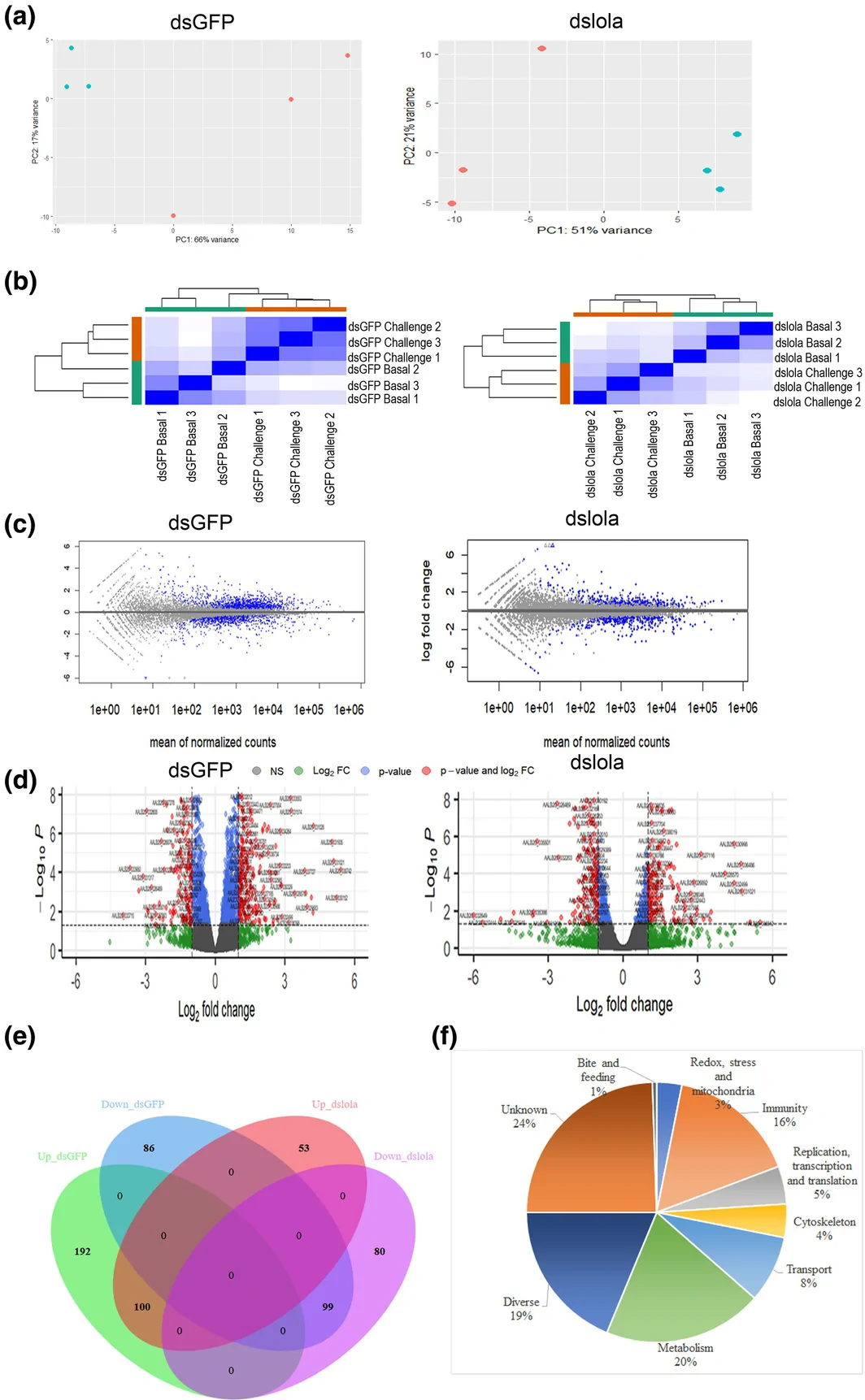
Transcriptome profiles produced by RNA‐seq. (a) PCA analysis showing the first principal component (PC1) on the *x*‐axis, while the second principal component (PC2) is on the *y*‐axis. Percentages denote the amount of variance explained by each different PC. PCA of the 3 basal dsGFP and 3 challenge dsGFP library replicates. Red circles represent basal replicates; blue circles represent challenge treatment replicates. (b) Sample Distance matrix plot. (c) MA‐Plots for the contrast basal vs. challenge showing normalized counts versus the log2(fold change). DE genes (*p*‐value <0.05) are coloured blue, and those that do not change their expression between comparison groups appear grey. Points that fall outside the window are plotted as open triangles, pointing up or down depending on whether their log2FoldChange value is positive or negative. (d) Volcano plots showing the points corresponding to genes identified as differentially expressed are highlighted in red (padj < 0.05 and log2foldChange > 1), green (log2foldChange > 1), blue (padj < 0.05), and those that do not change their expression between the comparison groups appear in grey. The data visualization and plotting were performed using DESeq2 in R. (e) A Venn diagram shows the number of individual genes that were induced or suppressed in response to the challenge and differentially expressed between the dsGFP‐ and dslola‐treated groups. Upregulated (log2FoldChange > 1, padj < 0.05) and downregulated (log2FoldChange < −1, padj <0.05). Numbers indicate gene transcripts upregulated/gene transcripts downregulated. (f) Distribution of the 192 transcripts that require *lola* expression for their upregulation according to their functional group: Bite and feeding (1); Redox, stress and mitochondria (6); Immunity (31); Replication, transcription and translation (9); Cytoskeleton (8); Transport (16); Metabolism (38); Diverse (36) and Unknown (47). The numbers indicate the percentage of genes in each functional group.

Differentially expressed genes were identified using DESeq2P. Principal component analysis (PCA) and a distance matrix were performed to validate the results. PCA indicated that 83% of the observed variance among the six library datasets was attributable to the presence/absence of challenge in the dsGFP group, with negligible variance attributable to biological replication. Similarly, PCA indicated that 72% of the observed variance across the six library datasets was attributable to the challenge in the dslola group (Figure 3a). A distance matrix is another way to examine how samples cluster. To do this, distances between groups are calculated, and colours are assigned, with the most similar samples displaying darker colours (Figure 3b). These comparative analyses of the 12 sequenced samples showed no differences in general properties between the basal and challenge biological replicates (silenced and non‐silenced). Therefore, differences between silenced and control samples are attributable to the *lola* silencing effect on the expression of multiple genes following an immune challenge. Next, the differentially expressed genes were identified and their potential functions investigated. Therefore, lola expression is required for the upregulation of multiple genes induced by an immune challenge.

### Genes induced by immune challenge and dependent on *lola* upregulation

To visualize the differential expression analysis, the mean expression (MeanBase) and log2 fold change were plotted in an MA plot, which shows the mean normalized read counts for each gene versus the base‐2 logarithm of the fold change. Differentially expressed transcripts are shown in blue (Figure 3c). Another plot that visualizes changes in differential expression is the volcano plot (Figure 3d), which shows the magnitude of transcript differences between two groups, with the most significant ones highlighted in red. The complete list of genes and transcripts that were differentially expressed with a padj < 0.05 and a log2 fold change >1 is provided in Table S3. The Venn diagram shows that 292 genes were significantly upregulated in the control group (dsGFP‐injected), indicating that the immune challenge activates their transcription. Only 192 of the genes upregulated by immune challenge require *lola* expression for transcriptional activation, whereas 100 do not (Figure 3e). Because the transcription levels of the differentially expressed genes depend on *lola* expression, we henceforth refer to them as *lola*‐regulated genes. Next, the functional categories of *lola*‐regulated genes induced by microbial challenge were investigated.

### *lola*‐regulated genes induced by microbial challenge

These differentially expressed genes (192) were classified into the following categories based on the putative function of the protein they encode. They belong to multiple functional clusters, including immunity (16%), redox, stress and mitochondria (3%), cytoskeletal (4%), metabolism (20%), transport (8%), replication, transcription and translation (5%), diverse (19%) and those of unknown function (24%) (Figure 3f).

**FIGURE 4:**
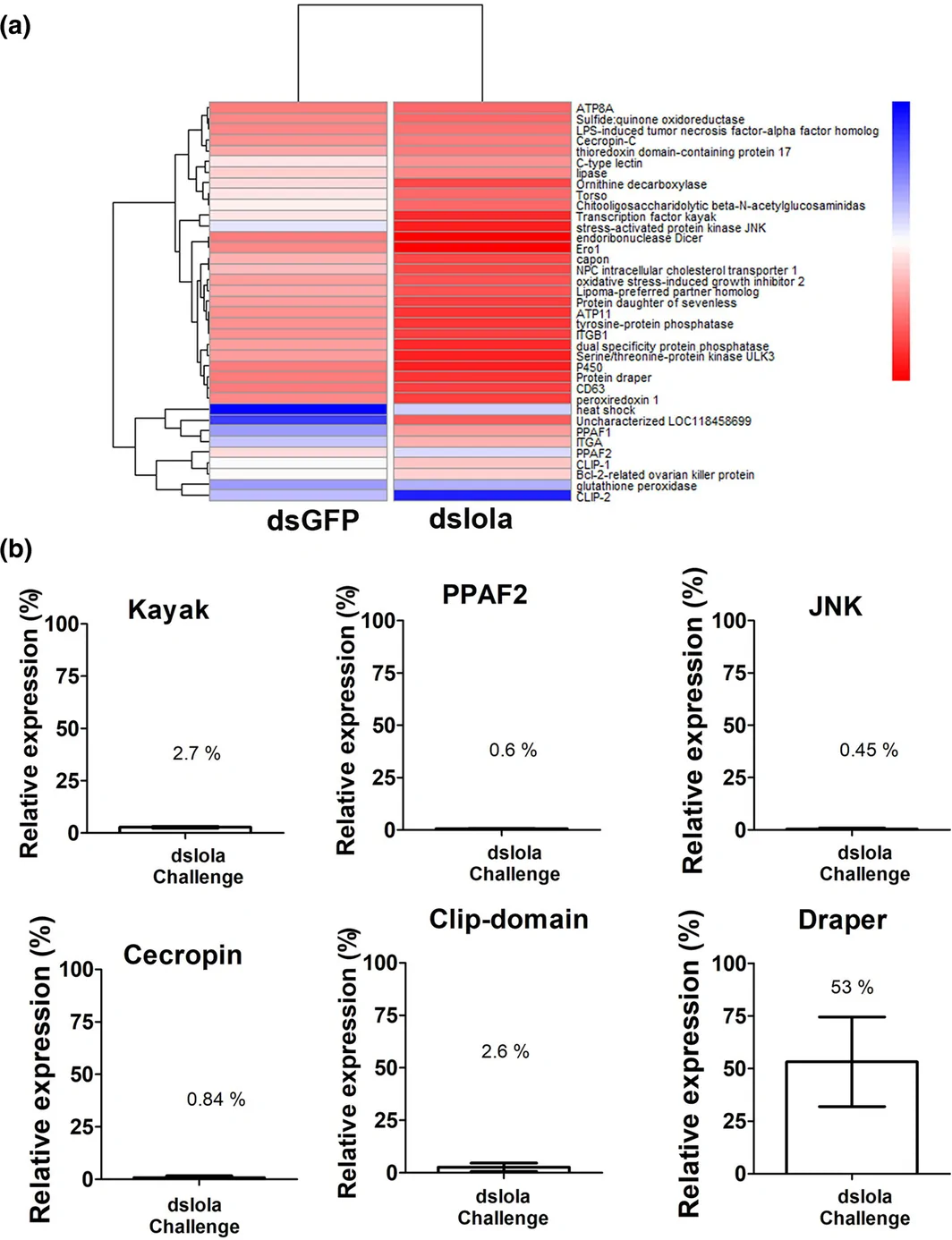
Downregulation of *lola* inhibits the immune challenge–induced upregulation of target genes. (a) The heat map shows 37 differentially expressed genes (rows) (log2‐fold change > 1) involved in the innate immune response and redox, stress, and mitochondria between the non‐silenced (dsGFP) and silenced (dslola) groups of differential expression genes visualized as a hierarchical clustering tree. The intensity of the colour represents the relative expression level. (b) Validation of RNA‐seq for individual genes: Each column and error bar represents the percentage of relative expression and the standard deviation of the mean compared with GFP dsRNA‐treated mosquito midguts at 24 h post dsRNA‐mediated silencing of *lola* and with bacterial challenge. The expression was determined by RT‐qPCR and normalized to the ribosomal gene S7. Significant differences between controls and silenced midguts are shown by asterisks (***p < 0.001).

Differential expression of some *lola*‐dependent genes was independently confirmed by RT‐qPCR. To further confirm the reliability and accuracy of the RNA‐seq differential expression results, six immunity‐related genes that were differentially expressed were selected for validation. Targets from this dataset were selected for validation based on raw padj and log2 fold change, as well as previously known immune involvement (Figure 4a), and their expression was evaluated by qRT‐PCR (Figure 4b). For this, midguts were dissected from five silenced adult *An. albimanus* female mosquitoes. Midguts from unsilenced mosquitoes were used as controls. All primer pairs used for differential gene expression confirmation were validated by dynamic range and melt‐curve analyses. (Figure S3). The expression patterns of these genes, as measured by qRT‐PCR, were highly concordant with those in the RNA‐seq data, indicating that the RNA‐seq data were reliable. These results confirm that *lola* attenuation precludes the upregulation of genes inducible by an immune challenge, leading to differential gene expression between attenuated and control mosquitoes.

### Immunity genes regulated by *lola*

RNAi‐mediated knockdown of *lola* showed that a total of 37 genes were differentially expressed and related to the innate immune response and redox, stress and mitochondria, 5 of the 31 bacterial‐responsive immune related genes are putatively linked to melanization (Table S4), including two clip‐domain serine proteases (CLIPs), two phenoloxidase‐activating factors (PPF1 and PPF2), one C‐type lectin and one antimicrobial peptide (AMP) cecropin C. Five are present in lysosomes: Niemann‐Pick disease type C1 (NPC1), member of the myeloid differentiation 2‐related lipid recognition protein (ML), NPC2, Chitooligosaccharidolytic beta‐N‐acetylglucosaminidase (HEXA_B), lipase enzyme (LIPA) and LPS‐induced TNF‐α factor (LITAF). Dicer‐2 gene, a key factor of the antiviral RNAi immune pathway (Kumar et al., 2018). Differential expression analysis revealed six JNK pathway members: JNK, Kayak (Kay), Buffy, Puc, Daughter of Sevenless (DOS) and Torso (TSL) were significantly downregulated in dslola‐treated mosquitoes. Four belong to the recognition group: Draper, two integrins and CD63. Two ATPases, Ornithine decarboxylase (ODC1) and dual specificity protein phosphatase (DUSP), are putatively linked to efferocytosis. Serine/threonine‐protein kinase (ULK3) is an autophagy pathway member. Proteins involved in oxidative stress, peroxiredoxin (PRDX1), glutathione peroxidase (GPX) and thioredoxin (TXD17), cytochrome P450 (CYP6), Sulfide: quinone oxidoreductase mitochondrial (Sqro), oxidative stress‐induced growth inhibitor 2 (OSGIN2) and capon as well as two putatively linked protein processing in endoplasmic reticulum heat shock/chaperone proteins (hsp70) and Ero 1 were significantly downregulated in dslola‐treated mosquitoes (Table S4).

### Molecular functions of *lola*‐dependent immunity genes

**FIGURE 5:**
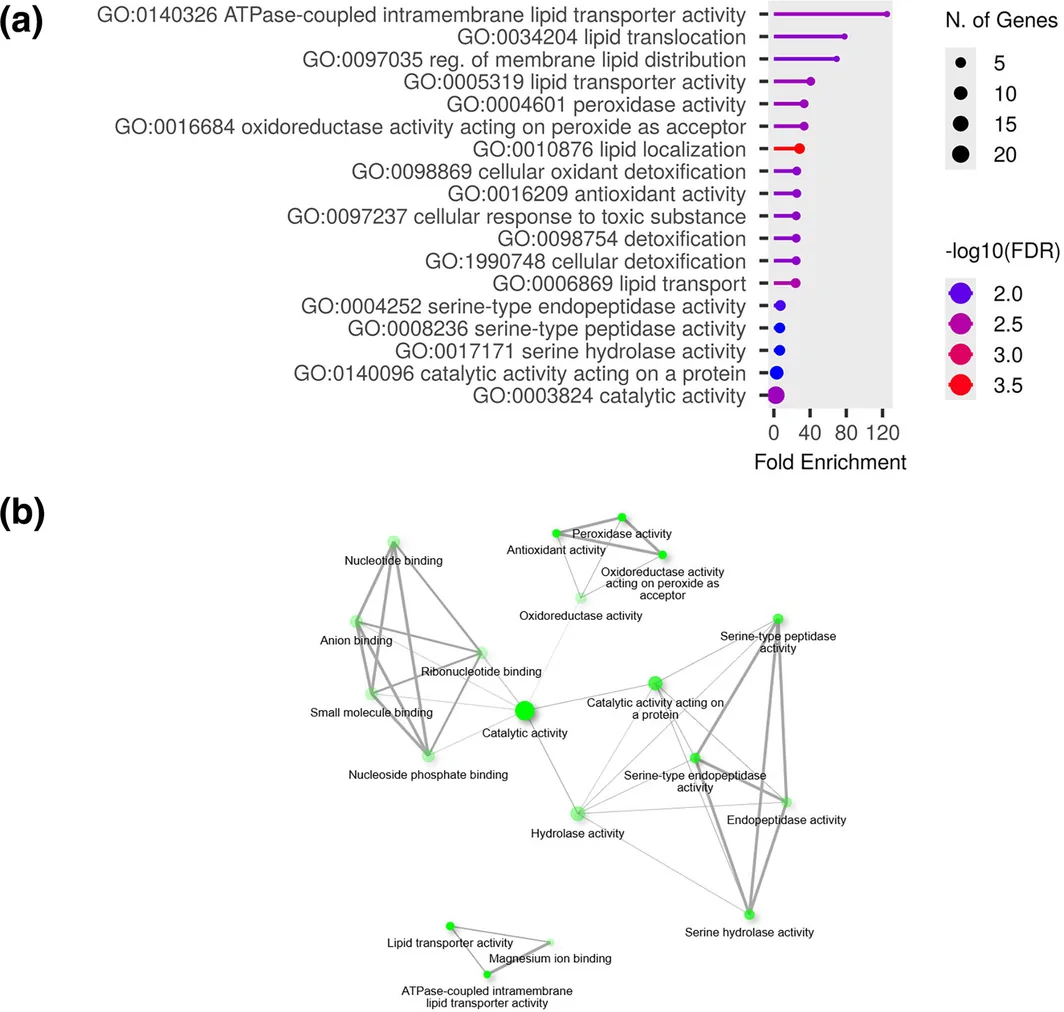
Immune‐associated genes that are differentially expressed in response to challenge and regulated by *lola* expression. (a) Gene ontology (GO) terms associated with differentially expressed genes (DEGs) from the biological functions and processes categories for differentially abundant 37 transcripts that were not induced in *lola*‐silenced mosquitoes with a significant level when these genes are mapped to the molecular pathways and functional categories in the available *Anopheles albimanus* databases. The circle size indicates the number of genes included in the category. (b) Network visualization. Graph obtained with ShinyGO.

Because the immune response is part of the phenotype of mosquito vector competence, the molecular functions of genes upregulated by *lola‐*dependent immune challenge were investigated. To determine the biological processes and molecular functions of the differentially expressed genes, gene set enrichment analysis and ontology were performed on 37 genes of particular interest and related to immunity (Figure 5a). The GO terms for biological processes (BP) were: GO:0003824 catalytic activity, GO:0016787 hydrolase activity, GO:0016491 oxidoreductase activity, GO:0004252 serine‐type endopeptidase activity, GO:0016209 antioxidant activity, GO:0005319 lipid transporter activity, GO:0098754 detoxification and GO:0009636 response to toxic substance.

Based on the annotated biological processes described by GO terms, we constructed a network to illustrate the links and significance of each GO term using ShinyGO (http://bioinformatics.sdstate.edu/go/) (Ge et al., 2020). The network shown in Figure 5b illustrates the 37 genes regulated by *lola* and enriched for BP. Their associated GO terms indicate an impact on catalytic activity, serine‐type endopeptidase activity and antioxidant activity.

KEGG analysis identified the most significantly enriched pathways as lysosomal function, efferocytosis and MAPK signalling. KEGG pathway analysis revealed proteins involved in various pathways; cecropin transcripts were enriched in midgut tissue after challenge, suggesting activation of the Imd and Toll pathways (Figure S5). We found that downstream transcription activators for JNK and Imd, that is, Kayak and JNK, respectively, were transcribed, confirming the conservation of these pathways across insects.

Our analysis of the pathway indicates that LITAF, LIPA, HEXA_B and CD63 are involved in the lysosomal pathway (Figure S6), which is necessary for the degradation of pathogens (Luzio et al., 2007). In addition, it revealed that proteins such as draper, ATPase, LIPA, ODC1 and DUSP, which are involved in the efferocytosis pathway (Figure S7), are also affected. Efferocytosis is the process by which dying target cells and pathogen‐infected cells are removed by phagocytic cells (Karaji & Sattentau, 2017). Integrins and Draper also contribute to efferocytosis in *Drosophila* (Dickson et al., 2026; Manaka et al., 2004).

KEGG pathway analysis identified transcripts representing the Wnt and Hippo signalling pathways. It has been shown that *Hippo signalling is also essential for maintaining immune system homeostasis in Drosophila* (Hong et al., 2018). Lipoma preferred partner (LPP), a focal adhesion protein with proline‐rich and LIM domains, was not induced in *lola*‐silenced mosquitoes. LPP is known to be involved in Hippo signalling, as is JNK. In dipterans, the adult midgut is a single epithelial layer with apical–basal polarity, which is essential for epithelial cell morphology and intestinal function. A previous study in *Drosophila* showed that Lola regulates midgut homeostasis and regeneration via noncanonical Hippo signalling, controlling organ size by balancing cell proliferation and death in intestinal stem cells (Hao et al., 2020). It has also been revealed that activation of WNT‐JNK signalling regulation is linked to planar cell polarity (Boutros et al., 1998). Therefore, Lola could regulate homeostasis through these two different pathways in mosquitoes.

Innate immune responses are the first line of defence in mosquitoes against the invasion of foreign microorganisms. CLIPs, a highly diverse group of proteolytic enzymes, play a crucial role in activating prophenoloxidase (Kanost & Jiang, 2015; Wang et al., 2021). Bacterial‐responsive genes, such as clip‐domain serine proteases, have been specifically linked to the Toll pathway (Kanost & Jiang, 2015). Cecropin has been linked to the Imd and Toll pathways (Moon et al., 2011; Zhang et al., 2017). Additionally, the present study revealed that *lola* regulates Dicer expression, a key gene in the defence against viral infections, and controls the expression of housekeeping genes (Campbell et al., 2008; Galiana‐Arnoux et al., 2006).

The MAPKs pathway is involved in physiological processes that influence metabolic and tissue homeostasis, cell death/survival and cell damage repair, and is required for immune responses (Dong et al., 2002). There are three major groups of MAPKs: the extracellular signal‐regulated protein kinases (ERKs), the p38 MAPKs, and the JNKs (Dong et al., 2002). Differential gene expression analysis revealed that *lola* expression is required for JNK expression. JNK signalling is involved in a wide range of biological processes in *Drosophila*, including embryonic development, apoptosis, stress response, cell proliferation and differentiation, longevity, phagocytosis and immunity (Kallio et al., 2005; Souvannaseng et al., 2018; Tafesh‐Edwards & Eleftherianos, 2020; Wang et al., 2003; Yan et al., 2022). The JNK pathway branches from the Imd pathway and phosphorylates a variety of transcription factors involved in aging and immunity in *Drosophila* (Park et al., 2004). We also observed a cluster of genes associated with the JNK pathway; among them, the transcription factor Kayak was not induced in *lola*‐silenced mosquitoes. Buffy and Puc were not induced in *lola*‐silenced mosquitoes. In *Drosophila*, the Fos homologue, Kay, is phosphorylated by JNK, which phosphorylates the Jun and Fos transcription factors, thereby generating a Jun/Fos dimer (AP‐1 complex) that activates transcription of target genes. JNK signalling is modulated by Puckered (Puc), a phosphatase that suppresses signalling by dephosphorylating JNK. Puckered is part of a negative feedback loop, because transcription of puc is regulated by the JNK pathway (Martin‐Blanco et al., 1998). While the Buffy protein may be antiapoptotic (Quinn et al., 2003).

Other transcripts that require *lola* and participate in the MAPK pathway encode for the DOS protein, which is involved in signalling from the Sevenless receptor tyrosine kinase (SEV) and TSL (Raabe, 2000; Raabe et al., 1996). The MAP kinase pathway is an important component in the regulation of antibacterial peptide expression. It has been reported that in the *Aedes albopictus* C6/36 cell line, LPS‐induced expression of antibacterial peptides is mediated by the JNK signalling pathway (Mizutani et al., 2003). The relationship between MAP kinase and Imd pathways in mosquitoes requires further investigation. Furthermore, it has been shown that the JNK pathway regulates caspase activation in *Plasmodium*‐invaded midgut cells; disruption of JNK signalling increases during the pathogen's invasion of mosquito tissues (Garver et al., 2013; Ramphul et al., 2015). This is consistent with the finding that JNK is essential for normal antimicrobial peptide release in mosquitoes (Kallio et al., 2005). JNK is involved in the autophagy pathway by inducing the expression of several autophagy‐related (ATG) genes, together with ULK3, which has also been linked to autophagy in *Drosophila* (Braden & Neufeld, 2016; Wu et al., 2009). Our results suggest that Lola may regulate immunity gene expression directly on effector genes, indirectly on components of immunity pathways, or both, as in the case of the MAPK pathways.

Differential gene expression RNA‐seq analyses presented here showed that among effector genes that require *lola* expression, NPC1 and NPC2 are among the most strongly expressed. These two effectors are a class of lipid‐binding proteins responsible for cholesterol transport and homeostasis pathway in *Drosophila melanogaster* (Garver & Heidenreich, 2002). It has been suggested that they may play a role in the Imd and Toll immune signalling pathways (Shi et al., 2012; Shimazu et al., 1999). KEGG pathway analysis revealed that NPC1 and NPC2 reside in and function closely together within the late endosomal/lysosomal compartment of cells, facilitating cholesterol trafficking and metabolism.

Additionally, an LITAF gene upregulated by immune challenge depends on *lola* expression. It has been shown that LITAF binds to the TNF‐α promoter in response to bacterial LPS stimulation and is induced in the midgut of *An. gambiae* in response to ookinete epithelial invasion (Smith et al., 2012; Tang et al., 2006).

The host recognizes pathogens through pattern recognition receptors (PRRs), which activate downstream immune signalling pathways, including the Toll and Imd pathways.

Draper is a single‐path membrane protein with EGF repeats. It appears to be a receptor for phagocytosis of apoptotic cells and *E. coli* by haemocytes in *Drosophila* (Sigle & Hillyer, 2018). Draper's expression is also enriched in the midgut (Hixson et al., 2022). In this study, Draper upregulation in response to immune challenge depended on *lola* expression. A group of potential cell‐surface PRRs involved in microbial recognition in *Drosophila* and *Anopheles gambiae* includes two integrins (Dupuy & Caron, 2008; Moita et al., 2006; Nonaka et al., 2013) and CD63, an exosome marker (Rana & Zoller, 2011).

The gene expression of various key components and signalling molecules in immune pathways appears to be regulated by *lola* expression, according to our results. A wide range of immune effectors downstream of PRR‐controlled and other recognition pathways includes antimicrobial peptides (AMPs), reactive oxygen species (ROS), reactive nitrogen species (RNS), prophenoloxidase (PPOs) and apoptosis‐ and phagocytosis‐related genes. ROS are potentially toxic to the host, so it is important that they be generated transiently and kept well localized. Host cells are protected from oxidative damage by enzymes that detoxify ROS, such as superoxide dismutase (SOD), which detoxifies superoxide; catalase, glutathione peroxidase (Gpx) and thioredoxin peroxidase, which detoxify hydrogen peroxide (H_2_O_2_). Thioredoxins are another class of antioxidant enzymes that participate in redox metabolism, and peroxiredoxins also regulate peroxide levels (Mates, 2000). The peroxiredoxins are a family of antioxidant peroxidases that reduce hydrogen peroxide and protect cells from nitrosative stress (Odnokoz et al., 2023). The components of redox/mitochondrial regulation regulate redox balance and cellular responses to oxidative stress. In the redox functional cluster, a gene encoding cytochrome P450 (CYP6) and mitochondrial Sulfide: quinone oxidoreductase (Sqro) showed lola‐dependent regulation (Kumar et al., 2022; Nauen et al., 2022). This dependence indicates that *lola* regulates detoxification in mosquitoes. Thioredoxins are also antioxidant enzymes involved in redox metabolism of anti‐P*lasmodium* (Jortzik & Becker, 2012). The transcripts of oxidative stress‐induced growth inhibitor 2 (OSGIN2) and capon, a nitric oxide synthase (Cui et al., 2025; Xie et al., 2023), also required *lola* expression.

Our studies revealed that three enzyme‐encoding genes (PRDX1, GPX and TXD17) involved in hydrogen peroxide detoxification depend on *lola* expression. Previous studies indicate that the JNK pathway is required for adult female mosquitoes to survive oxidative stress and to regulate gene expression, including Gpx expression and heat shock protein levels (Jaramillo‐Gutierrez et al., 2010). PRDX1 has been reported to contribute to H_2_O_2_‐induced activation of the p38 signalling pathway (Barata & Dick, 2020). The JNK pathway also induces the expression of the engulfment receptor Draper in mosquitoes and *Drosophila* (Macdonald et al., 2013; Weavers et al., 2016; Yan et al., 2022). Furthermore, we evaluated their expression by qRT‐PCR, and the expression patterns obtained were highly consistent with those in the RNA‐seq data. Our results are consistent with *An. gambiae* transcriptomic data showing that expressed AMPs, receptor genes and chitinases are robustly expressed in the midguts and strongly expressed in the proventriculi (Hixson et al., 2022).

### Some genes from other functional clusters are also regulated by *lola*

Some metabolism‐related genes are also regulated by *lola*; 20% of DE genes are involved in metabolism (Table S4). For example, *lola‐*regulated transcripts are potentially related to lipid metabolism and digestion, including lipases, three ceramides that are central molecules in sphingolipid metabolism, a sphingomyelin synthase, and other lipases. In protein metabolism, three peptidases, non‐CLIP serine endopeptidases, trypsins and chymotrypsin‐related proteases, metalloproteinases (MMPs) and collagenase are involved in extracellular matrix remodelling and tissue repair. MMPs are enzymes that degrade collagen. In carbohydrate metabolism, among them are collagenase, glucose‐6‐phosphate dehydrogenase and inositol‐trisphosphate 3‐kinase. Genes regulated by *lola* were also found in nucleotide metabolism (adenylyl cyclase), xenobiotic metabolism (trans‐1,2‐dihydrobenzene‐1,2‐diol dehydrogenase), nitrogen metabolism (carbonic anhydrase 2) and hormone biosynthesis (cholesterol 7‐desaturase). Chitin is essential for maintaining the barrier function of the peritrophic matrix in the mosquito midgut (Kelkenberg et al., 2015), and two putative chitinases were also found here to be *lola*‐dependent.

Cytoskeletal and other related genes are regulated by *lola*; 4% (8 genes) of *lola*‐regulated transcripts are involved in the cytoskeleton (Table S4), mainly gelsolin and two myosins. Defects in gelsolin expression affect the barrier function in the *Aedes aegypti* midgut (Cui et al., 2024).

Five percent of lola‐regulated transcripts (including 9 genes) are involved in replication, transcription and translation. The main ones are the Krueppel transcription factor, translation initiation factor 2 (IF2), an essential GTP/GDP‐binding protein that interacts specifically with the ribosomal P site for translation initiation (Marzi et al., 2003), and histone H1 (Table S4). In addition, other membrane transport genes (including 16 genes) that required lola expression, among them two ATPases, three sugar transport genes, four ion transporters and six ABC (ATP‐binding cassette) transporters, were identified as *lola*‐regulated in this study, including ABCG4, ABCC4, ABCC4, ABCA3, ABCC10 and ABCG1 (Table S4). Recently, the role of ABC transporters in insect immunity has also been described in the midgut of *Aedes aegypti* (Kumar et al., 2025).

An additional group of diverse genes, each with multiple functions, is also regulated by *lola*. This group showed significant enrichment for genes (36 in total) involved in the regulation of various cellular processes, an insulin‐like growth factor‐binding protein, two adhesion molecules and two G protein‐coupled receptors, among others (Table S4).

### Molecular functions of other *lola*‐dependent genes

Apart from immunity genes, other biological function gene ontologies (GOs) were enriched among *lola*‐dependent genes. These Biological Process GOs regulated by *lola* were GO:0006030 chitin metabolic process, GO:1901564 organonitrogen compound metabolic process, GO:0006665 sphingolipid metabolic process, GO:0030154 cell differentiation, GO:0048729 tissue morphogenesis and GO:0048468 cell development (Figure S4A). We analyse the network of the complete list of differentially expressed genes, their associated GO terms, and suggest impacts on morphogenesis, ATP hydrolysis activity, chitin metabolic process and integral component of membrane (Figure S4B).

### Lola regulates the detoxification process in the midgut

**FIGURE 6:**
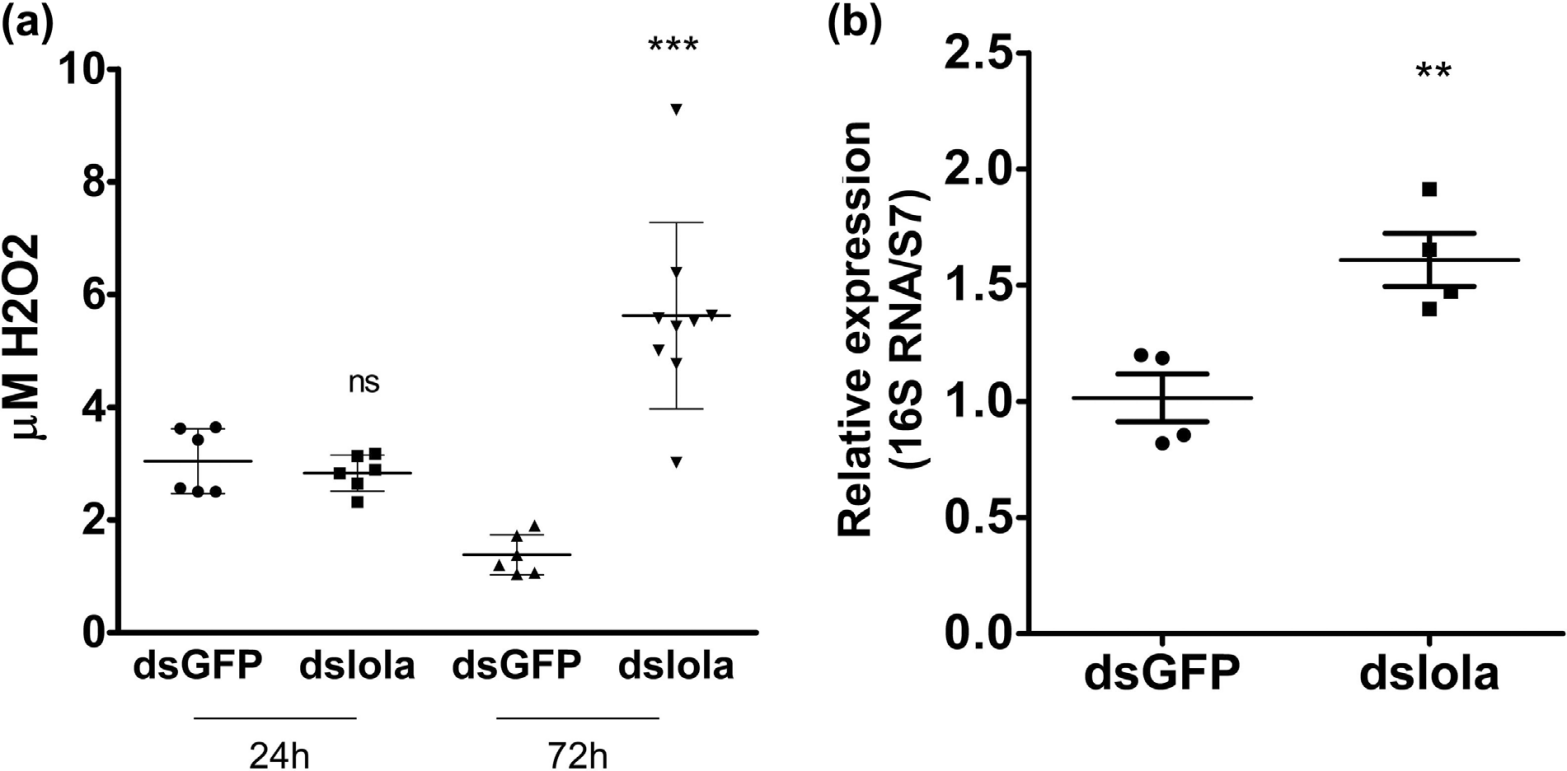
Lola's silencing in the midgut increases bacterial load and ROS levels. (a) Measurement of H_2_O_2_ in midgut at 24 and 72 h post‐injection with dslola or dsGFP. At least two biological replicates, each with a pool of 5 midguts from each condition, were used. Significance was determined by Student's *t*‐test ***p < 0.0001, and ns (not significant) were indicated with each set of quantification. (b) Relative quantification of 16S rRNA in midgut. Mosquitoes were injected with dsGFP (controls) or dslola (silenced) RNA, and relative 16S rRNA levels were analysed in their midguts 2 days post‐injection. Ribosomal gene S7 was used as an internal control. Two biological replicates, each with a pool of 5 midguts, were used. Significance was determined by Student's *t*‐test, **p < 0.001.

RNA‐seq analysis of midgut gene expression described above showed lower PRDX1, GPX and TXD17 expression in the midguts of *lola*‐silenced mosquitoes. To determine whether those changes modify midgut redox metabolism, we measured H_2_O_2_ released by epithelial cells. Figure 6a shows that *lola* knockdown increased the amount of hydrogen peroxide leaked into the supernatant. These data confirm that attenuation of *lola* expression by dslola injection reduces peroxidase activity and alters redox metabolism in the midgut. These metabolic alterations are consistent with the strong effect of *lola* attenuation on the detoxification‐related gene expression observed in *lola‐attenuated* mosquito midguts. These results confirm the participation of lola in the mosquito innate immune response against microbial invaders.

### Lola regulates the bacterial homeostasis in the midgut

Mosquito midgut microbes are involved in digestion, metabolism, detoxification and immunity (Minard et al., 2013). Since *lola* attenuation compromises mosquito survival against a live bacterial challenge and regulates antimicrobial defence genes, we hypothesized that *lola* expression might also regulate bacterial homeostasis in the midgut. To test this hypothesis, the load of endogenous bacteria in the midgut of *lola*‐attenuated mosquitoes was evaluated. The analyses of 16S rRNA levels of endogenous bacteria in the midguts of *lola*‐silenced and control mosquitoes indicated that their levels increased ~0.5‐fold in *lola*‐attenuated mosquitoes 48 h after dsRNA injection (Figure 6b). RNAi‐mediated silencing of the *lola* gene significantly enhanced the growth of midgut bacteria. These results indicate a negative correlation between *lola* expression and midgut bacterial growth. Other mosquito genes are involved in maintaining bacterial homeostasis. For instance, in *Anopheles stephensi* mosquitoes, similar results as those presented here were obtained by regulating the expression of heme peroxidase 2 (Kakani et al., 2020). The significant changes in bacterial load in the midgut of *lola*‐attenuated mosquitoes suggest that Lola contributes to vector competence by maintaining midgut homeostasis. These results also show the involvement of *lola* in the defence against microbial invaders.

In conclusion, here we provided further evidence supporting the hypothesis that the Lola transcription factor is involved in the mosquito innate immune response (Perez‐Zamorano et al., 2017). In this study, we showed that the *lola* ortholog in mosquitoes participates in the regulation of immune signalling pathways and other biological processes such as biogenesis, development, response to stimulus and gene expression, and that these biological functions are conserved between mosquitoes and *D. melanogaster (*Bass et al., 2007; Ryan et al., 2020; Silva et al., 2016). Therefore, the fact that the *lola* expression‐attenuated phenotype affects biological processes previously known to be targets of Lola in *D. melanogaster* supports the conclusion that the silenced gene is the lola ortholog in *An. albimanus*. In addition, this study showed that several bacterial challenge‐induced genes previously reported to be involved in the anti‐*Plasmodium* immune response are also dependent on *lola* expression. Therefore, we suggest that *lola* is a gene involved in vector competence, as many genes involved in the anti‐*Plasmodium* response are regulated by *lola* expression.

We identified clusters of genes that were differentially expressed in immune challenged mosquitoes with and without attenuation of *lola* gene expression. These gene clusters represent different *lola* expression‐dependent immunity pathways in the mosquito midgut, including efferocytosis, lysosomes and phagosomes, autophagy, peroxisomes, JNK signalling, protein processing in the endoplasmic reticulum and apoptosis. These data confirm that *lola* expression in the mosquito midgut is required to regulate activation of the innate immune response.

These results support the hypothesis that the transcription factor encoded by *lola* participates in the molecular mechanisms of mosquito innate immune response. It is important to note that, according to the data presented in this work, *lola* regulates immune networks and pathways upstream of effector immunity genes. It is then expected that changes in *lola* expression and/or functional mutations will substantially affect the immune phenotype of mosquitoes, thereby modifying the expression of multiple immunity genes. The data presented in this work warrant further studies to identify and analyse *lola* regulatory elements. We suggest that polymorphic sites within *lola* regulatory elements could affect its expression levels, the immunity phenotype and, therefore, the mosquito vector competence phenotype. The results presented here warrant adding Lola to the network of known transcription factors that regulate the mosquito's innate immune response, thereby updating current models.

The genus *Anopheles*, which includes major malaria vectors such as *An*. *gambiae*, *An. stephensi*, *An. maculatus*, *An. sinensis* and *An. darlingi*, belongs to the family Culicidae of dipterans and diverged from Muscomorpha, to which *Drosophila* belongs, 260 million years ago. The conservation of the *lola* gene between *An. albimanus* and *Drosophila* suggest it is an ancestral gene. Here, evidence was provided for the conservation of some *lola* biological functions between *An. albimanus* and *D. melanogaster*. At least one putative ortholog of *lola* exists in each of the 16 *Anopheles* species and two *Aedes* species with sequenced and annotated genomes in VectorBase (Giraldo‐Calderon et al., 2015; Neafsey et al., 2015). We suggest, then, that the conclusions reached here regarding the relevance of *lola* to *An. albimanus* innate immune response and oxidative stress response apply to Culicinae. The relevance of *lola* to the vector competence phenotype of anopheline vectors may affect malaria parasite transmission and, therefore, the public health of exposed populations worldwide.

## AUTHOR CONTRIBUTIONS

**Heidi Espadas‐Álvarez:** Conceptualization (supporting); data curation (equal); formal analysis (equal); funding acquisition (equal); investigation (equal); methodology (equal); project administration (equal); resources (supporting); validation (supporting); writing – original draft (equal); writing – review and editing (equal). **Verónica Valverde‐Garduño:** Conceptualization (lead); data curation (equal); formal analysis (equal); funding acquisition (equal); investigation (equal); methodology (equal); project administration (equal); resources (lead); supervision (lead); validation (lead); writing – original draft (equal); writing – review and editing (equal).

## FUNDING INFORMATION

This work was supported by Grant CF‐2023‐I‐2327 to HE‐A and VV‐G provided by the National Council of Science and Technology (CONACYT), México: Postdoctoral Fellowship to HE‐A: CVU 421099.

## CONFLICT OF INTEREST STATEMENT

The authors declare no conflicts of interest.

## Supporting information


**Figure S1.** Molecular analysis of *lola* silencing in *Anopheles albimanus*. Effect of dslola microinjections on *lola* mRNA expression in the mosquito midgut. qRT‐PCR was performed and compared with the dsGFP group (control). Silencing efficiency of *lola* 24 and 72 h post microinjection. The relative expression of *lola* in dslola versus dsGFP‐injected (control) mosquitoes was calculated through the method 2^−∆∆CT^ (Livak & Schmittgen, 2001) and statistically analysed by Student's *t*‐test. Percent knockdown was calculated by subtracting the normalized 2^−∆∆CT^ (defined by the level of expression for dsGFP) and multiplying by 100.
**Figure S2.** Box plot of data quality before and after preprocessing. Representative images of quality values per base of the sample reads (A) before and (B) after initial quality processing with FastQC.
**Figure S3.** Linear dynamic range and efficiency of the RT‐qPCR assays. Standard curves were generated by serially diluting cDNA. (A) Primers for *lola* gene (efficiency 110%), (B) primers for Clip‐domain gene (efficiency 93.2%), (C) primers for kayak gene (efficiency 109.6%), (D) primers for Draper gene (efficiency 95.7%), (E) primers for Cecropin gene (efficiency 97.9%), (F) primers for PPAF2 gene (efficiency 99.6%) and (G) primers for JNK gene (efficiency 156.6%). The logarithm to base 10 (log) of the cDNA copy number versus the cycle threshold (CT) value is represented. Each dot represents the Cq‐value from two replicates, and the lines represent the best‐fitting log‐linear regression models.
**Figure S4.** Overview of the functional categories of all differentially expressed genes (DEGs) in *lola*‐silenced mosquitoes. (A) GO enrichment analysis: GO terms from the biological functions and processes categories for differentially abundant 192 transcripts that were not induced by immune challenge in *lola*‐silenced mosquitoes. The circle size indicates the number of genes included in the category. (B) Network visualization. Graph obtained with ShinyGO.
**Figure S5.** Genes for components of the Toll and Imd signalling pathways that are differentially expressed in *lola* KD mosquitoes. The components of this signalling pathway that were identified as differentially expressed in our analysis are highlighted in red. Pathway painting was performed with log2‐fold change and the KEGG ID for each gene using SRplot. This colour‐coded map of DEGs corresponds to aali04624 in the KEGG database, corresponding to the signalling pathway model for *Anopheles albimanus*. The JNK pathway branches out from the Imd pathway.
**Figure S6.** Genes for components of the lysosomal signalling pathway that are differentially expressed in *lola* KD mosquitoes. The components of this signalling pathway that were identified as differentially expressed in our analysis are highlighted in red. Pathway painting was performed with log2‐fold change and KEGG ID for each gene using SRplot. This colour‐coded map of DEGs corresponds to aali04142 in the KEGG database, corresponding to the signalling pathway model for *Anopheles albimanus*.
**Figure S7.** Genes for components of the efferocytosis signalling pathway that are differentially expressed in *lola* KD mosquitoes. The components of this signalling pathway that were identified as differentially expressed in our analysis are highlighted in red. Pathway painting was performed with log2‐fold change and KEGG ID for each gene using SRplot. This colour‐coded map of DEGs corresponds to aali04148 in the KEGG database, corresponding to the signalling pathway model for *Anopheles albimanus*.

**Table S1.** Summary of RNA sequencing data generated.

**Table S2.** Primers used for RTqPCR assays.

**Table S3.** Complete list of genes and transcripts that were differentially expressed.

**Table S4.** Categories of genes that were differentially expressed.

**Table S5.** Raw sequence data SRA accession numbers.

## Data Availability

The data supporting the findings of this study have been deposited in NCBI's Gene Expression Omnibus (Clough et al., 2023; Edgar et al., 2002) and are accessible through GEO Series accession number GSE317204: https://www.ncbi.nlm.nih.gov/geo/query/acc.cgi?acc=GSE317204 (Valverde‐Garduño & Espadas‐Álvarez, 2026). The raw sequence data accession numbers and direct links are listed in Table S5.

## References

[imb70051-bib-0001] Abraham, E.G. & Jacobs‐Lorena, M. (2004) Mosquito midgut barriers to malaria parasite development. Insect Biochemistry and Molecular Biology, 34(7), 667–671. 15242707 10.1016/j.ibmb.2004.03.019

[imb70051-bib-0002] Bahia, A.C. , Kubota, M.S. , Tempone, A.J. , Araujo, H.R. , Guedes, B.A. , Orfano, A.S. et al. (2011) The JAK‐STAT pathway regulates *Plasmodium vivax* load during the early stages of Anopheles aquasalis infection. PLoS Neglected Tropical Diseases, 5, e1317. 22069502 10.1371/journal.pntd.0001317 PMC3206008

[imb70051-bib-0003] Barata, A.G. & Dick, T.P. (2020) A role for peroxiredoxins in H_2_O_2_‐ and MEKK‐ dependent activation of the p38 signaling pathway. Redox Biology, 28, 101340. 31629169 10.1016/j.redox.2019.101340 PMC6807362

[imb70051-bib-0004] Barillas‐Mury, C. , Han, Y.S. , Seeley, D. & Kafatos, F.C. (1999) *Anopheles gambiae* Ag‐STAT, a new insect member of the STAT family, is activated in response to bacterial infection. The EMBO Journal, 18(4), 959–967. 10022838 10.1093/emboj/18.4.959 PMC1171188

[imb70051-bib-0005] Barletta, A.B.F. , Saha, B. , Trisnadi, N. , Talyuli, O.A.C. , Raddi, G. & Barillas‐Mury, C. (2022) Hemocyte differentiation to the megacyte lineage enhances mosquito immunity against *Plasmodium* . eLife, 11, e81116. 36052991 10.7554/eLife.81116 PMC9545522

[imb70051-bib-0006] Bass, B.P. , Cullen, K. & McCall, K. (2007) The axon guidance gene lola is required for programmed cell death in the *Drosophila ovary* . Developmental Biology, 304, 771–785. 17336958 10.1016/j.ydbio.2007.01.029 PMC1905497

[imb70051-bib-0007] Bolger, A.M. , Lohse, M. & Usadel, B. (2014) Trimmomatic: a flexible trimmer for Illumina sequence data. Bioinformatics, 30, 2114–2120. 24695404 10.1093/bioinformatics/btu170 PMC4103590

[imb70051-bib-0008] Boutros, M. , Paricio, N. , Strutt, D.I. & Mlodzik, M. (1998) Disheveled activates JNK and discriminates between JNK pathways in planar polarity and wingless signaling. Cell, 94, 109–118. 9674432 10.1016/s0092-8674(00)81226-x

[imb70051-bib-0009] Braden, C.R. & Neufeld, T.P. (2016) Atg1‐independent induction of autophagy by the *Drosophila* Ulk3 homolog, ADUK. The FEBS Journal, 283, 3889–3897. 27717182 10.1111/febs.13906 PMC5123689

[imb70051-bib-0010] Campbell, C.L. , Black, W.C., 4th , Hess, A.M. & Foy, B.D. (2008) Comparative genomics of small RNA regulatory pathway components in vector mosquitoes. BMC Genomics, 18(9), 425. 10.1186/1471-2164-9-425 PMC2566310 18801182

[imb70051-bib-0011] Catteruccia, F. & Levashina, E.A. (2009) RNAi in the malaria vector, *Anopheles gambiae* . Métodos de Biología Molecular, 555, 63–75. 10.1007/978-1-60327-295-7_5 19495688

[imb70051-bib-0012] Clayton, A.M. , Dong, Y. & Dimopoulos, G. (2014) The Anopheles innate immune system in the defense against malaria infection. Journal of Innate Immunity, 6(2), 169–181. 23988482 10.1159/000353602 PMC3939431

[imb70051-bib-0013] Clough, E. , Barrett, T. , Wilhite, S.E. , Ledoux, P. , Evangelista, C. , Kim, I.F. et al. (2023) NCBI GEO: Archive for gene expression and epigenomics data sets: 23‐year update. Nucleic Acids Research, 52(D1), D138–D144. 10.1093/nar/gkad965 PMC10767856 37933855

[imb70051-bib-0014] Compton, A. , Liang, J. , Chen, C. , Lukyanchikova, V. , Qi, Y. , Potters, M. et al. (2020) The beginning of the end: a chromosomal assembly of the new world malaria mosquito ends with a novel telomere. G3 (Bethesda), 10, 3811–3819. 32883756 10.1534/g3.120.401654 PMC7534423

[imb70051-bib-0015] Crowner, D. , Madden, K. , Goeke, S. & Giniger, E. (2002) Lola regulates midline crossing of CNS axons in *Drosophila* . Development, 129, 1317–1325. 11880341 10.1242/dev.129.6.1317

[imb70051-bib-0016] Cui, Q.F. , Liu, C. , Dong, X.M. & Liu, Z.Q. (2025) Exploring the biological functions and disease implications of OSGINs: a journey from discovery to clinical relevance. Biochemical Pharmacology, 237, 116921. 40199404 10.1016/j.bcp.2025.116921

[imb70051-bib-0017] Cui, Y. , Megawati, D. , Lin, J. , Rehard, D.G. , Grant, D.G. , Liu, P. et al. (2024) Cytoskeleton‐associated gelsolin responds to the midgut distention process in saline meal‐fed *Aedes aegypti* and affects arbovirus dissemination from the midgut. The FASEB Journal, 38(14), e23764. 39042395 10.1096/fj.202302684RR PMC11268798

[imb70051-bib-0018] Dickson, B.H. , Tasnim, T. , Nicholson, R.A. , Stanlake, N. , Lam, A.L. & Vrieze, A.M. (2026) MERTK coordinates efferocytosis by regulating integrin localization and activation. Journal of Cell Science, 139(6), jcs264792. 41716147 10.1242/jcs.264792 PMC13070255

[imb70051-bib-0019] Dobin, A. , Davis, C.A. , Schlesinger, F. , Drenkow, J. , Zaleski, C. , Jha, S. et al. (2013) STAR: ultrafast universal RNA‐seq aligner. Bioinformatics, 29, 15–21. 23104886 10.1093/bioinformatics/bts635 PMC3530905

[imb70051-bib-0020] Dong, C. , Davis, R.J. & Flavell, R.A. (2002) MAP kinases in the immune response. Annual Review of Immunology, 20, 55–72. 10.1146/annurev.immunol.20.091301.131133 11861597

[imb70051-bib-0021] Dupuy, A.G. & Caron, E. (2008) Integrin‐dependent phagocytosis: spreading from microadhesion to new concepts. Journal of Cell Science, 121, 1773–1783. 18492791 10.1242/jcs.018036

[imb70051-bib-0022] Edgar, R. , Domrachev, M. & Lash, A.E. (2002) Gene expression omnibus: NCBI gene expression and hybridization array data repository. Nucleic Acids Research, 30(1), 207–210. 11752295 10.1093/nar/30.1.207 PMC99122

[imb70051-bib-0023] Ehret, G.B. , Reichenbach, P. , Schindler, U. , Horvath, C.M. , Fritz, S. , Nabholz, M. et al. (2001) DNA binding specificity of different STAT proteins. Comparison of in vitro specificity with natural target sites. American Society of Biological Chemists, 276, 6675–6688. 10.1074/jbc.M001748200 11053426

[imb70051-bib-0024] Galiana‐Arnoux, D. , Dostert, C. , Schneemann, A. , Hoffmann, J.A. & Imler, J.‐L. (2006) Essential function in vivo for Dicer‐2 in host defense against RNA viruses in *Drosophila* . Nature Immunology, 7, 590–597. 16554838 10.1038/ni1335

[imb70051-bib-0025] Garver, L.S. , De Almeida Oliveira, G. & Barillas‐Mury, C. (2013) The JNK pathway is a key mediator of *Anopheles gambiae* antiplasmodial immunity. PLoS Pathogens, 9, e1003622. 24039583 10.1371/journal.ppat.1003622 PMC3764222

[imb70051-bib-0026] Garver, W.S. & Heidenreich, R.A. (2002) The Niemann‐Pick C proteins and trafficking of cholesterol through the late endosomal/lysosomal system. Current Molecular Medicine, 2(5), 485–505. 12125814 10.2174/1566524023362375

[imb70051-bib-0027] Gates, M.A. , Kannan, R. & Giniger, E. (2011) A genome‐wide analysis reveals that the *Drosophila* transcription factor Lola promotes axon growth, in part, by suppressing the expression of the actin nucleation factor Spire. Neural Development, 6, 37. 22129300 10.1186/1749-8104-6-37 PMC3262752

[imb70051-bib-0028] Ge, S.X. , Jung, D. & Yao, R. (2020) ShinyGO: a graphical gene‐set enrichment tool for animals and plants. Bioinformatics, 36, 2628–2629. 31882993 10.1093/bioinformatics/btz931 PMC7178415

[imb70051-bib-0029] Gendrin, M. , Turlure, F. , Rodgers, F.H. , Cohuet, A. , Morlais, I. & Christophides, G.K. (2017) The peptidoglycan recognition proteins PGRPLA and PGRPLB regulate anopheles immunity to bacteria and affect infection by plasmodium. Journal of Innate Immunity, 9, 333–342. 28494453 10.1159/000452797 PMC5569699

[imb70051-bib-0030] Giniger, E. , Tietje, K. , Jan, L.Y. & Jan, Y.N. (1994) lola encodes a putative transcription factor required for axon growth and guidance in *Drosophila* . Development, 120, 1385–1398. 8050351 10.1242/dev.120.6.1385

[imb70051-bib-0031] Giraldo‐Calderon, G.I. , Emrich, S.J. , Maccallum, R.M. , Maslen, G. , Dialynas, E. , Topalis, P. et al. (2015) VectorBase: an updated bioinformatics resource for invertebrate vectors and other organisms related to human diseases. Nucleic Acids Research, 43, D707–D713. 25510499 10.1093/nar/gku1117 PMC4383932

[imb70051-bib-0032] Goeke, S. , Greene, E.A. , Grant, P.K. , Gates, M.A. , Crowner, D. , Aigaki, T. et al. (2003) Alternative splicing of lola generates 19 transcription factors controlling axon guidance in *Drosophila* . Nature Neuroscience, 6, 917–924. 12897787 10.1038/nn1105

[imb70051-bib-0033] Gupta, L. , Molina‐Cruz, A. , Kumar, S. , Rodrigues, J. , Dixit, R. , Zamora, R.E. et al. (2009) The STAT pathway mediates late‐phase immunity against *Plasmodium* in the mosquito *Anopheles gambiae* . Cell Host & Microbe, 5, 498–507. 19454353 10.1016/j.chom.2009.04.003 PMC2701194

[imb70051-bib-0034] Hao, X. , Wang, S. , Lu, Y. , Yu, W. , Li, P. , Jiang, D. et al. (2020) Lola regulates *Drosophila* adult midgut homeostasis via non‐canonical Hippo signaling. eLife, 14(9), e47542. 10.7554/eLife.47542 PMC7299341 31934851

[imb70051-bib-0035] Hardy, J.L. , Houk, E.J. , Kramer, L.D. & Reeves, W.C. (1983) Intrinsic factors affecting vector competence of mosquitoes for arboviruses. Annual Review of Entomology, 28, 229–262. 10.1146/annurev.en.28.010183.001305 6131642

[imb70051-bib-0036] Hixson, B. , Bing, X.L. , Yang, X. , Bonfini, A. , Nagy, P. & Buchon, N. (2022) A transcriptomic atlas of *Aedes aegypti* reveals detailed functional organization of major body parts and gut regional specializations in sugar‐fed and blood‐fed adult females. eLife, 26(11), e76132. 10.7554/eLife.76132 PMC9113746 35471187

[imb70051-bib-0037] Hong, L. , Li, X. , Zhou, D. , Geng, J. & Chen, L. (2018) Role of Hippo signaling in regulating immunity. Cellular & Molecular Immunology, 15(12), 1003–1009. 29568120 10.1038/s41423-018-0007-1 PMC6269503

[imb70051-bib-0038] Jaramillo‐Gutierrez, G. , Molina‐Cruz, A. , Kumar, S. & Barillas‐Mury, C. (2010) The *Anopheles gambiae* oxidation resistance 1 (OXR1) gene regulates expression of enzymes that detoxify reactive oxygen species. PLoS One, 5, e11168. 20567517 10.1371/journal.pone.0011168 PMC2887368

[imb70051-bib-0039] Jortzik, E. & Becker, K. (2012) Thioredoxin and glutathione systems in *Plasmodium* falciparum. International Journal of Medical Microbiology, 302, 187–194. 22939033 10.1016/j.ijmm.2012.07.007

[imb70051-bib-0040] Kakani, P. , Gupta, L. & Kumar, S. (2020) Heme‐peroxidase 2, a peroxinectin‐like gene. Regulates bacterial homeostasis in *Anopheles stephensi* midgut. Frontiers in Physiology, 8(11), 572340. 10.3389/fphys.2020.572340 PMC7506126 33013485

[imb70051-bib-0041] Kallio, J. , Leinonen, A. , Ulvila, J. , Valanne, S. , Ezekowitz, R.A. & Ramet, M. (2005) Functional analysis of immune response genes in *Drosophila* identifies JNK pathway as a regulator of antimicrobial peptide gene expression in S2 cells. Microbes and Infection/Institut Pasteur, 7, 811–819. 10.1016/j.micinf.2005.03.014 15890554

[imb70051-bib-0042] Kanehisa, M. & Goto, S. (2000) KEGG: Kyoto encyclopedia of genes and genomes. Nucleic Acids Research, 28, 27–30. 10592173 10.1093/nar/28.1.27 PMC102409

[imb70051-bib-0043] Kanost, M.R. & Jiang, H. (2015) Clip‐domain serine proteases as immune factors in insect hemolymph. Current Opinion in Insect Science, 11, 47–55. 26688791 10.1016/j.cois.2015.09.003 PMC4680995

[imb70051-bib-0044] Karaji, N. & Sattentau, Q.J. (2017) Efferocytosis of pathogen‐infected cells. Frontiers in Immunology, 22(8), 1863. 10.3389/fimmu.2017.01863 PMC5743670 29312342

[imb70051-bib-0045] Kelkenberg, M. , Odman‐Naresh, J. , Muthukrishnan, S. & Merzendorfer, H. (2015) Chitin is a necessary component to maintain the barrier function of the peritrophic matrix in the insect midgut. Insect Biochemistry and Molecular Biology, 56, 21–28. 25449129 10.1016/j.ibmb.2014.11.005

[imb70051-bib-0046] Khan, M.B. , Liew, J.W. , Leong, C.S. & Lau, Y.L. (2016) Role of NF‐kbeta factor Rel2 during *Plasmodium* falciparum and bacterial infection in *Anopheles dirus* . Parasites and Vectors, 9, 525. 27688040 10.1186/s13071-016-1810-0 PMC5041562

[imb70051-bib-0047] Kleino, A. , Valanne, S. , Ulvila, J. , Kallio, J. , Myllymaki, H. , Enwald, H. et al. (2005) Inhibitor of apoptosis 2 and TAK1‐binding protein are components of the *Drosophila* Imd pathway. The EMBO Journal, 24, 3423–3434. 16163390 10.1038/sj.emboj.7600807 PMC1276168

[imb70051-bib-0048] Kumar, A. , Srivastava, P. , Sirisena, P. , Dubey, S.K. , Kumar, R. , Shrinet, J. et al. (2018) Mosquito innate immunity. Insects, 9, 95. 30096752 10.3390/insects9030095 PMC6165528

[imb70051-bib-0049] Kumar, R. , Landry, A.P. , Guha, A. , Vitvitsky, V. , Lee, H.J. , Seike, K. et al. (2022) A redox cycle with complex II prioritizes sulfide quinone oxidoreductase‐dependent H2S oxidation. The Journal of Biological Chemistry, 298(1), 101435. 34808207 10.1016/j.jbc.2021.101435 PMC8683732

[imb70051-bib-0050] Kumar, V. , Garg, S. , Sisodia, D. , Gupta, L. , Kumar, S. & Saxena, V. (2025) Midgut immune profiling and functional characterization of Aedes aegypti ABC transporter gene(s) using systemic and local bacterial challenges. Parasites & Vectors, 18(1), 34. 39891271 10.1186/s13071-025-06658-6 PMC11786363

[imb70051-bib-0051] Liao, Y. , Smyth, G.K. & Shi, W. (2014) featureCounts: an efficient general purpose program for assigning sequence reads to genomic features. Bioinformatics, 30, 923–930. 24227677 10.1093/bioinformatics/btt656

[imb70051-bib-0052] Luzio, J.P. , Pryor, P.R. & Bright, N.A. (2007) Lysosomes: fusion and function. Nature Reviews. Molecular Cell Biology, 8(8), 622–632. 17637737 10.1038/nrm2217

[imb70051-bib-0053] Macdonald, J.M. , Doherty, J. , Hackett, R. & Freeman, M.R. (2013) The c‐Jun kinase signaling cascade promotes glial engulfment activity through activation of Draper and phagocytic function. Cell Death and Differentiation, 20, 1140–1148. 23618811 10.1038/cdd.2013.30 PMC3741495

[imb70051-bib-0054] Manaka, J. , Kuraishi, T. , Shiratsuchi, A. , Nakai, Y. , Higashida, H. , Henson, P. et al. (2004) Draper‐mediated and phosphatidylserine‐independent phagocytosis of apoptotic cells by *Drosophila hemocytes*/macrophages. The Journal of Biological Chemistry, 279(46), 48466–48476. 15342648 10.1074/jbc.M408597200

[imb70051-bib-0055] Martin‐Blanco, E. , Gampel, A. , Ring, J. , Virdee, K. , Kirov, N. , Tolkovsky, A.M. et al. (1998) Puckered encodes a phosphatase that mediates a feedback loop regulating JNK activity during dorsal closure in *Drosophila* . Genes and Development, 12, 557–570. 9472024 10.1101/gad.12.4.557 PMC316530

[imb70051-bib-0056] Marzi, S. , Knight, W. , Brandi, L. , Caserta, E. , Soboleva, N. , Hill, W.E. et al. (2003) Ribosomal localization of translation initiation factor IF2. RNA, 9(8), 958–969. 12869707 10.1261/rna.2116303 PMC1370462

[imb70051-bib-0057] Mates, J.M. (2000) Effects of antioxidant enzymes in the molecular control of reactive oxygen species toxicology. Toxicology, 153, 83–104. 11090949 10.1016/s0300-483x(00)00306-1

[imb70051-bib-0058] Meredith, J.M. , Munks, R.J. , Grail, W. , Hurd, H. , Eggleston, P. & Lehane, M.J. (2006) A novel association between clustered NF‐kappaB and C/EBP binding sites is required for immune regulation of mosquito Defensin genes. Insect Molecular Biology, 15(4), 393–401. 16907826 10.1111/j.1365-2583.2006.00635.x PMC1602061

[imb70051-bib-0059] Michel, K. & Kafatos, F.C. (2005) Mosquito immunity against *Plasmodium* . Insect Biochemistry and Molecular Biology, 35, 677–689. 15894185 10.1016/j.ibmb.2005.02.009

[imb70051-bib-0060] Minard, G. , Mavingui, P. & Moro, C. (2013) Diversity and function of bacterial microbiota in the mosquito holobiont. Parasites & Vectors, 6, 146. 23688194 10.1186/1756-3305-6-146 PMC3667145

[imb70051-bib-0061] Mizutani, T. , Kobayashi, M. , Eshita, Y. , Inanami, O. , Yamamori, T. , Goto, A. et al. (2003) Characterization of JNK‐like protein derived from a mosquito cell line, C6/36. Insect Molecular Biology, 12, 61–66. 12542636 10.1046/j.1365-2583.2003.00387.x

[imb70051-bib-0062] Moita, L.F. , Vriend, G. , Mahairaki, V. , Louis, C. & Kafatos, F.C. (2006) Integrins of *Anopheles gambiae* and a putative role of a new beta integrin, BINT2, in phagocytosis of *E. coli* . Insect Biochemistry and Molecular Biology, 36, 282–290. 16551542 10.1016/j.ibmb.2006.01.004

[imb70051-bib-0063] Moon, A.E. , Walker, A.J. & Goodbourn, S. (2011) Regulation of transcription of the *Aedes albopictus* cecropin A1 gene: a role for p38 mitogen‐activated protein kinase. Insect Biochemistry and Molecular Biology, 41, 628–636. 21501684 10.1016/j.ibmb.2011.04.001

[imb70051-bib-0064] Nauen, R. , Bass, C. , Feyereisen, R. & Vontas, J. (2022) The role of cytochrome P450s in insect toxicology and resistance. Annual Review of Entomology, 67, 105–124. 10.1146/annurev-ento-070621-061328 34590892

[imb70051-bib-0065] Neafsey, D.E. , Waterhouse, R.M. , Abai, M.R. , Aganezov, S.S. , Alekseyev, M.A. , Allen, J.E. et al. (2015) Mosquito genomics. Highly evolvable malaria vectors: the genomes of 16 anopheles mosquitoes. Science, 347, 1258522. 25554792 10.1126/science.1258522 PMC4380271

[imb70051-bib-0066] Nonaka, S. , Nagaosa, K. , Mori, T. , Shiratsuchi, A. & Nakanishi, Y. (2013) Integrin alphaPS3/betanu‐mediated phagocytosis of apoptotic cells and bacteria in *Drosophila* . The Journal of Biological Chemistry, 288, 10374–10380. 23426364 10.1074/jbc.M113.451427 PMC3624420

[imb70051-bib-0067] Odnokoz, O. , Earland, N. , Badinloo, M. , Klichko, V.I. , Benes, J. , Orr, W.C. et al. (2023) Peroxiredoxins play an important role in the regulation of immunity and aging in *Drosophila* . Antioxidants (Basel), 12, 1616. 37627611 10.3390/antiox12081616 PMC10451867

[imb70051-bib-0068] Ohsako, T. , Horiuchi, T. , Matsuo, T. , Komaya, S. & Aigaki, T. (2003) *Drosophila* lola encodes a family of BTB‐transcription regulators with highly variable C‐terminal domains containing zinc finger motifs. Gene, 311, 59–69. 12853139 10.1016/s0378-1119(03)00554-7

[imb70051-bib-0069] Park, J.M. , Brady, H. , Ruocco, M.G. , Sun, H. , Williams, D. , Lee, S.J. et al. (2004) Targeting of TAK1 by the NF‐kappa B protein relish regulates the JNK‐mediated immune response in *Drosophila* . Genes and Development, 18, 584–594. 15037551 10.1101/gad.1168104 PMC374239

[imb70051-bib-0108] Pérez‐Zamorano, B ., Rosas‐Madrigal, S ., Lozano, OAM ., Castillo Méndez, M ., Valverde‐Garduño, V . (2017) Identification of cis‐regulatory sequences reveals potential participation of lola and Deaf1 transcription factors in Anopheles gambiae innate immune response. PLoS One, 12(10), e0186435. Available from: 10.1371/journal.pone.0186435. 29028826 PMC5640250

[imb70051-bib-0070] Qayum, A.A. & Telang, A. (2011) A protocol for collecting and staining hemocytes from the yellow fever mosquito *Aedes aegypti* . Journal of Visualized Experiments, 51, 2772. 10.3791/2772 PMC3197126 21633325

[imb70051-bib-0071] Quinn, L. , Coombe, M. , Mills, K. , Daish, T. , Colussi, P. , Kumar, S. et al. (2003) Buffy, a *Drosophila* Bcl‐2 protein, has anti‐apoptotic and cell cycle inhibitory functions. The EMBO Journal, 22, 3568–3579. 12853472 10.1093/emboj/cdg355 PMC165625

[imb70051-bib-0072] Raabe, T. (2000) The sevenless signaling pathway: variations of a common theme. Biochimica et Biophysica Acta, 1496, 151–163. 10771085 10.1016/s0167-4889(00)00020-3

[imb70051-bib-0073] Raabe, T. , Riesgo‐Escovar, J. , Liu, X. , Bausenwein, B.S. , Deak, P. , Maröy, P. et al. (1996) DOS, a novel pleckstrin homology domain‐containing protein required for signal transduction between sevenless and Ras1 in *Drosophila* . Cell, 85(6), 911–920. 8681385 10.1016/s0092-8674(00)81274-x

[imb70051-bib-0074] Ramphul, U.N. , Garver, L.S. , Molina‐Cruz, A. , Canepa, G.E. & Barillas‐Mury, C. (2015) *Plasmodium falciparum* evades mosquito immunity by disrupting JNK‐mediated apoptosis of invaded midgut cells. Proceedings of the National Academy of Sciences of the United States of America, 112, 1273–1280. 25552553 10.1073/pnas.1423586112 PMC4321252

[imb70051-bib-0075] Rana, S. & Zoller, M. (2011) Exosome target cell selection and the importance of exosomal tetraspanins: a hypothesis. Biochemical Society Transactions, 39, 559–562. 21428939 10.1042/BST0390559

[imb70051-bib-0076] Ryan, S.M. , Wildman, K. , Oceguera‐Perez, B. , Barbee, S. , Mortimer, N.T. & Vrailas‐Mortimer, A.D. (2020) Evolutionarily conserved transcription factors drive the oxidative stress response in *Drosophila* . The Journal of Experimental Biology, 223, jeb221622. 32532866 10.1242/jeb.221622 PMC7391405

[imb70051-bib-0078] Schmittgen, T.D. & Livak, K.J. (2008) Analyzing real‐time PCR data by the comparative C(T) method. Nature Protocols, 3, 1101–1108. 18546601 10.1038/nprot.2008.73

[imb70051-bib-0079] Sharma, P. , Rani, J. , Chauhan, C. , Kumari, S. , Tevatiya, S. , De Das, T. et al. (2020) Altered gut microbiota and immunity define plasmodium vivax survival in Anopheles stephensi. Frontiers in Immunology, 11, 609. 32477320 10.3389/fimmu.2020.00609 PMC7240202

[imb70051-bib-0080] Shi, X.Z. , Zhong, X. & Yu, X.Q. (2012) *Drosophila melanogaster* NPC2 proteins bind bacterial cell wall components and may function in immune signal pathways. Insect Biochemistry and Molecular Biology, 42, 545–556. 22580186 10.1016/j.ibmb.2012.04.002 PMC3358802

[imb70051-bib-0081] Shimazu, R. , Akashi, S. , Ogata, H. , Nagai, Y. , Fukudome, K. , Miyake, K. et al. (1999) MD‐2, a molecule that confers lipopolysaccharide responsiveness on toll‐like receptor 4. The Journal of Experimental Medicine, 189(11), 1777–1782. 10359581 10.1084/jem.189.11.1777 PMC2193086

[imb70051-bib-0082] Shin, S.W. , Kokoza, V. , Bian, G. , Cheon, H.M. , Kim, Y.J. & Raikhel, A.S. (2005) REL1, a homolog of drosophila dorsal, regulates toll antifungal immune pathway in the female mosquito *Aedes aegypti* . Journal of Biological Chemistry, 280, 16499–16507. 15722339 10.1074/jbc.M500711200

[imb70051-bib-0083] Sigle, L.T. & Hillyer, J.F. (2018) Eater and draper are involved in the periostial hemocyte immune response in the mosquito *Anopheles gambiae* . Insect Molecular Biology, 27, 429–438. 29520896 10.1111/imb.12383

[imb70051-bib-0084] Silva, D. , Olsen, K.W. , Bednarz, M.N. , Droste, A. , Lenkeit, C.P. , Chaharbakhshi, E. et al. (2016) Regulation of gonad morphogenesis in *Drosophila melanogaster* by BTB family transcription factors. PLoS One, 11, e0167283. 27898696 10.1371/journal.pone.0167283 PMC5127561

[imb70051-bib-0085] Silverman, N. , Zhou, R. , Erlich, R.L. , Hunter, M. , Bernstein, E. , Schneider, D. et al. (2003) Immune activation of Nf‐Kappab and Jnk requires *drosophila* Tak1. The Journal of Biological Chemistry, 278, 48928–48934. 14519762 10.1074/jbc.M304802200

[imb70051-bib-0086] Sinka, M.E. , Rubio‐Palis, Y. , Manguin, S. , Patil, A.P. , Temperley, W.H. , Gething, P.W. et al. (2010) The dominant *anopheles* vectors of human malaria in the Americas: occurrence data, distribution maps, and bionomic precis. Parasites and Vectors, 3, 72. 20712879 10.1186/1756-3305-3-72 PMC2936890

[imb70051-bib-0087] Smith, R.C. , Eappen, A.G. , Radtke, A.J. & Jacobs‐Lorena, M. (2012) Regulation of anti‐*plasmodium* immunity by a LITAF‐like transcription factor in the malaria vector *Anopheles gambiae* . PLoS Pathogen, 8, e1002965. 23093936 10.1371/journal.ppat.1002965 PMC3475675

[imb70051-bib-0088] Souvannaseng, L. , Hun, L.V. , Baker, H. , Klyver, J.M. , Wang, B. , Pakpour, N. et al. (2018) Inhibition of JNK signaling in the Asian malaria vector Anopheles stephensi extends mosquito longevity and improves resistance to *plasmodium* falciparum infection. PLoS Pathogens, 14, e1007418. 30496310 10.1371/journal.ppat.1007418 PMC6264519

[imb70051-bib-0089] Tafesh‐Edwards, G. & Eleftherianos, I. (2020) JNK signaling in *drosophila* immunity and homeostasis. Immunology Letter, 226, 7–11. 10.1016/j.imlet.2020.06.017 32598968

[imb70051-bib-0090] Tang, D. , Chen, M. , Huang, X. , Zhang, G. , Zeng, L. , Wu, S. et al. (2023) SRplot: a free online platform for data visualization and graphing. PLoS One, 18, e0294236. 37943830 10.1371/journal.pone.0294236 PMC10635526

[imb70051-bib-0091] Tang, X. , Metzger, D. , Leeman, S. & Amar, S. (2006) LPS‐induced TNF‐alpha factor (LITAF)‐deficient mice express reduced LPS‐induced cytokine: evidence for LITAF‐dependent LPS signaling pathways. Proceedings of the National Academy of Sciences of the United States of America, 103, 13777–13782. 16954198 10.1073/pnas.0605988103 PMC1560089

[imb70051-bib-0092] Terradas, G. , Novelo, M. , Metz, H. , Brustolin, M. & Rasgon, J.L. (2023) *Anopheles albimanus* is a potential alphavirus vector in the Americas. The American Journal of Tropical Medicine and Hygiene, 108, 412–423. 36535260 10.4269/ajtmh.22-0417 PMC9896319

[imb70051-bib-0093] Tikhe, C.V. & Dimopoulos, G. (2021) Mosquito antiviral immune pathways. Developmental and Comparative Immunology, 116, 103964. 33301792 10.1016/j.dci.2020.103964

[imb70051-bib-0094] Valverde‐Garduño, V. & Espadas‐Álvarez, H.G. (2026) *Anopheles albimanus* lola knockdown immune response differential transcriptome: effect of *Anopheles albimanus* lola knockdown on the gene expression profile of the midgut in mosquitoes treated with an immune challenge. Gene Expression Omnibus; Accession GSE 317204. Available from: https://www.ncbi.nlm.nih.gov/geo/query/acc.cgi?acc=GSE317204

[imb70051-bib-0095] Wang, H.C. , Wang, Q.H. , Bhowmick, B. , Li, Y.X. & Han, Q. (2021) Functional characterization of two clip domain serine proteases in innate immune responses of *Aedes aegypti* . Parasites & Vectors, 14, 1–13. 34819136 10.1186/s13071-021-05091-9 PMC8611957

[imb70051-bib-0096] Wang, M.C. , Bohmann, D. & Jasper, H. (2003) JNK signaling confers tolerance to oxidative stress and extends lifespan in *Drosophila* . Developmental Cell, 5, 811–816. 14602080 10.1016/s1534-5807(03)00323-x

[imb70051-bib-0097] Wang, Y.H. , Wang, J.M. , Jiang, H. & Zhou, Z. (2013) Research advances on immune mechanism against pathogens in mosquitoes. Chinese Journal of Vector Biology and Control, 24, 477–482.

[imb70051-bib-0098] Weavers, H. , Evans, I.R. , Martin, P. & Wood, W. (2016) Corpse engulfment generates a molecular memory that primes the macrophage inflammatory response. Cell, 165, 1658–1671. 27212238 10.1016/j.cell.2016.04.049 PMC4912690

[imb70051-bib-0099] WHO . (2024) World malaria report 2024. Geneva: World Health Organization.

[imb70051-bib-0100] Wu, H. , Wang, M.C. & Bohmann, D. (2009) JNK protects *Drosophila* from oxidative stress by trancriptionally activating autophagy. Mechanisms of Development, 126, 624–637. 19540338 10.1016/j.mod.2009.06.1082 PMC2750887

[imb70051-bib-0101] Xie, W. , Xing, N. , Qu, J. , Liu, D. & Pang, Q. (2023) The physiological function of nNOS‐associated CAPON proteins and the roles of CAPON in diseases. International Journal of Molecular Sciences, 24, 15808. 37958792 10.3390/ijms242115808 PMC10647562

[imb70051-bib-0102] Yan, Y. , Sigle, L.T. , Rinker, D.C. , Estevez‐Lao, T.Y. , Capra, J.A. & Hillyer, J.F. (2022) The immune deficiency and c‐Jun N‐terminal kinase pathways drive the functional integration of the immune and circulatory systems of mosquitoes. Open Biology, 12, 220111. 36069078 10.1098/rsob.220111 PMC9449813

[imb70051-bib-0103] Zhang, R. , Zhu, Y. , Pang, X. , Xiao, X. , Zhang, R. & Cheng, G. (2017) Regulation of antimicrobial peptides in *Aedes aegypti* Aag2 cells. Frontiers Cellular and Infection Microbiology, 7, 22. 10.3389/fcimb.2017.00022 PMC5291090 28217557

[imb70051-bib-0104] Zhang, W. , Tettamanti, G. , Bassal, T. , Heryanto, C. , Eleftherianos, I. & Mohamed, A. (2021) Regulators and signalling in insect antimicrobial innate immunity: functional molecules and cellular pathways. Cellular Signaling, 83, 110003. 10.1016/j.cellsig.2021.110003 33836260

[imb70051-bib-0105] Zhang, W. , Wang, Y. , Long, J. , Girton, J. , Johansen, J. & Johansen, K.M. (2003) A developmentally regulated splice variant from the complex lola locus encoding multiple different zinc finger domain proteins interacts with the chromosomal kinase JIL‐1. The Journal of Biological Chemistry, 278, 11696–11704. 12538650 10.1074/jbc.M213269200

[imb70051-bib-0106] Zhao, T. , Xiao, Y. , Huang, B. , Ran, M.J. , Duan, X. , Wang, Y.F. et al. (2022) A dual role of lola in *Drosophila* ovary development: regulating stem cell niche establishment and repressing apoptosis. Cell Death and Disease, 13, 756. 36056003 10.1038/s41419-022-05195-9 PMC9440207

