# Supplementary material for: Malaria mosquito antimicrobial defence requires immunity and detoxification gene regulation by Lola
Source: Insect Mol Biol. 2026 Jun 20;35(5):576–93. doi: 10.1111/imb.70051 (PMC13551534; doi:10.1111/imb.70051)
Supplement: Supplementary file 1 [file IMB-35-576-s001.pdf]

Table S2. Primers used for RTqPCR assays

Name	Primes 5'- 3'
Aal_lola F	AGGATGAGAATGTGGAGG
Aal_lola R	TGAGGAAGTCGGAAGAAG
AalCec1 F	GTGGACGCTGGTTTTCTC
AalCec1 R	TTATTTGCCAAGTGCCTT C
S7 1F	CAAGAAGGCCATCGTCATCTAC
S7 1R	CCGAGAACTTCTTCTCCAGTTC
Aal_Clip F2	CGAAAAATGGAGTGCAAAGAGATGG
Aal_Clip R2	ATAGCATCCAACCGCCAGG
Aal_PPAF2 F2	ATGGCCAAGAGCGAACAC
Aal_PPAF2 R2	TGGTTCCGATATGCTTCTTTTC
Aal_Kayak F1	GGTGGTAATGGTGGTCTC
Aal_Kayak R1	TTCCAGGCTGTCGCTTTG
Aal_JNK F1	GATCGCTTCCCCCTAACACAC
Aal_JNK R1	ACAGCTTCTTCACCGCCAG
Aal_Drpr F1	CGACTGGAAAAACACCGAAA
Aal_Drpr R1	TGTGAACAGGGCGATGAG
16S RNA F	TTG GAG AGT TTG ATC CTG GCT C (Sharma et. Al 2020)
16S RNA R	ACG TCA TCC CCA CCT TCC TC (Sharma et. Al 2020)
