# Supplementary material for: Malaria mosquito antimicrobial defence requires immunity and detoxification gene regulation by Lola
Source: Insect Mol Biol. 2026 Jun 20;35(5):576–93. doi: 10.1111/imb.70051 (PMC13551534; doi:10.1111/imb.70051)
Supplement: Supplementary file 2 [file IMB-35-576-s003.pdf]

Table S5. Raw sequence data SRA accession numbers

Title	SRA Accession	URL link	Reference
dsGFP-Basal rep 1	SRX31886214	https://www.ncbi.nlm.nih.gov/geo/query/acc.cgi?acc=GSM9466950	1
dsGFP-Basal rep 2	SRX31886215	https://www.ncbi.nlm.nih.gov/geo/query/acc.cgi?acc=GSM9466951	2
dsGFP-Basal rep 3	SRX31886216	https://www.ncbi.nlm.nih.gov/geo/query/acc.cgi?acc=GSM9466952	3
dsGFP-Challenge rep 1	SRX31886217	https://www.ncbi.nlm.nih.gov/geo/query/acc.cgi?acc=GSM9466953	4
dsGFP-Challenge rep 2	SRX31886218	https://www.ncbi.nlm.nih.gov/geo/query/acc.cgi?acc=GSM9466954	5
dsGFP-Challenge rep 3	SRX31886219	https://www.ncbi.nlm.nih.gov/geo/query/acc.cgi?acc=GSM9466955	6
dslola-Basal rep 1	SRX31886220	https://www.ncbi.nlm.nih.gov/geo/query/acc.cgi?acc=GSM9466956	7
dslola-Basal rep 2	SRX31886221	https://www.ncbi.nlm.nih.gov/geo/query/acc.cgi?acc=GSM9466957	8
dslola-Basal rep 3	SRX31886222	https://www.ncbi.nlm.nih.gov/geo/query/acc.cgi?acc=GSM9466958	9
dslola- Challenge rep 1	SRX31886223	https://www.ncbi.nlm.nih.gov/geo/query/acc.cgi?acc=GSM9466959	10
dslola- Challenge rep 2	SRX31886224	https://www.ncbi.nlm.nih.gov/geo/query/acc.cgi?acc=GSM9466960	11
dslola- Challenge rep 3	SRX31886225	https://www.ncbi.nlm.nih.gov/geo/query/acc.cgi?acc=GSM9466961	12

References

1. Valverde-Garduño V, Espadas-Álvarez HG. dsGFP-Basal rep 1. Sequence Read Archive. Bethesda (MD): National Center for Biotechnology Information; 2026. Accession No. SRX31886214; 2026. Available from: https://www.ncbi.nlm.nih.gov/geo/query/acc.cgi?acc=GSM9466950.

2. Valverde-Garduño V, Espadas-Álvarez HG. dsGFP-Basal rep 2. Sequence Read Archive. Bethesda (MD): National Center for Biotechnology Information; 2026. Accession No. SRX31886215; 2026. Available from: https://www.ncbi.nlm.nih.gov/geo/query/acc.cgi?acc=GSM9466951

3. Valverde-Garduño V, Espadas-Álvarez HG. dsGFP-Basal rep 3. Sequence Read Archive. Bethesda (MD): National Center for Biotechnology Information; 2026. Accession No. SRX31886216; 2026. Available from: https://www.ncbi.nlm.nih.gov/geo/query/acc.cgi?acc=GSM9466952

4. Valverde-Garduño V, Espadas-Álvarez HG. dsGFP-Challenge rep 1. Sequence Read Archive. Bethesda (MD): National Center for Biotechnology Information; 2026. Accession No. SRX31886217; 2026. Available from: https://www.ncbi.nlm.nih.gov/geo/query/acc.cgi?acc=GSM9466953

5. Valverde-Garduño V, Espadas-Álvarez HG. dsGFP-Challenge rep 2. Sequence Read Archive. Bethesda (MD): National Center for Biotechnology Information; 2026. Accession No. SRX31886218; 2026. Available from: https://www.ncbi.nlm.nih.gov/geo/query/acc.cgi?acc=GSM9466954

6. Valverde-Garduño V, Espadas-Álvarez HG. dsGFP-Challenge rep 3. Sequence Read Archive. Bethesda (MD): National Center for Biotechnology Information; 2026. Accession No. SRX31886219; 2026. Available from: https://www.ncbi.nlm.nih.gov/geo/query/acc.cgi?acc=GSM9466955

7. Valverde-Garduño V, Espadas-Álvarez HG. dslola-Basal rep 1. Sequence Read Archive. Bethesda (MD): National Center for Biotechnology Information; 2026. Accession No. SRX31886220; 2026. Available from: https://www.ncbi.nlm.nih.gov/geo/query/acc.cgi?acc=GSM9466956

8. Valverde-Garduño V, Espadas-Álvarez HG. dslola-Basal rep 2. Sequence Read Archive. Bethesda (MD): National Center for Biotechnology Information; 2026. Accession No. SRX31886221; 2026. Available from: https://www.ncbi.nlm.nih.gov/geo/query/acc.cgi?acc=GSM9466957

9. Valverde-Garduño V, Espadas-Álvarez HG. dslola-Basal rep 3. Sequence Read Archive. Bethesda (MD): National Center for Biotechnology Information; 2026. Accession No. SRX31886222; 2026. Available from: https://www.ncbi.nlm.nih.gov/geo/query/acc.cgi?acc=GSM9466958

10. Valverde-Garduño V, Espadas-Álvarez HG. dslola- Challenge rep 1. Sequence Read Archive. Bethesda (MD): National Center for Biotechnology Information; 2026. Accession No. SRX31886223; 2026. Available from: https://www.ncbi.nlm.nih.gov/geo/query/acc.cgi?acc=GSM9466959

11. Valverde-Garduño V, Espadas-Álvarez HG. dslola- Challenge rep 2. Sequence Read Archive. Bethesda (MD): National Center for Biotechnology Information; 2026. Accession No. SRX31886224; 2026. Available from: https://www.ncbi.nlm.nih.gov/geo/query/acc.cgi?acc=GSM9466960

12. Valverde-Garduño V, Espadas-Álvarez HG. dslola- Challenge rep 3. Sequence Read Archive. Bethesda (MD): National Center for Biotechnology Information; 2026. Accession No. SRX31886225; 2026. Available from: https://www.ncbi.nlm.nih.gov/geo/query/acc.cgi?acc=GSM9466961
