# Supplementary material for: Malaria mosquito antimicrobial defence requires immunity and detoxification gene regulation by Lola
Source: Insect Mol Biol. 2026 Jun 20;35(5):576–93. doi: 10.1111/imb.70051 (PMC13551534; doi:10.1111/imb.70051)
Supplement: Supplementary file 3 [file IMB-35-576-s006.pdf]

Table S1. Summary of RNA-sequencing data generated

#	Biological treatment	Raw reads	Clean reads	Uniquely mapped reads	mapped reads %	Total alignments
1	dsGFP-Basal	13313091	12987708	11842746	91.18	21913992 (85.5%)
2	dsGFP-Basal	13262543	12865191	12005117	93.31	22096852 (86.3%)
3	dsGFP-Basal	11822004	11470441	10470699	91.28	19368085 (85.2%)
1	dsGFP-Challenge	13108188	12731956	11864358	93.19	21804075 (86.4%)
2	dsGFP- Challenge	13143953	12762546	11774320	92.26	21478918 (85.0%)
3	dsGFP- Challenge	12334517	12012949	10987108	91.46	20127062 (85.1%)
1	dslola-Basal	11981038	11653101	11055317	94.87	20342692 (87.3%)
2	dslola-Basal	12026052	11667822	10845215	92.95	20122951 (86.6%)
3	dslola-Basal	12699622	12354526	11322278	91.64	20950611 (85.8%)
1	dslola- Challenge	12135659	11802155	10975982	93	20106583 (85.9%)
2	dslola- Challenge	13371104	12916041	11857725	91.81	21633040 (84.6%)
3	dslola- Challenge	12779495	12326434	11349757	2.08	20760015 (84.8%)
