# Supplementary material for: Malaria mosquito antimicrobial defence requires immunity and detoxification gene regulation by Lola
Source: Insect Mol Biol. 2026 Jun 20;35(5):576–93. doi: 10.1111/imb.70051 (PMC13551534; doi:10.1111/imb.70051)
Supplement: Supplementary file 4 [file IMB-35-576-s004.docx]

**Supplementary information**

**Figure S1. Molecular analysis of *lola* silencing in *An. albimanus*.** Effect of dslola microinjections on *lola* mRNA expression in the mosquito midgut. qRT-PCR was performed and compared to the dsGFP group (control). Silencing efficiency of *lola* 24 and 72 h post microinjection. The relative expression of *lola* in dslola versus dsGFP-injected (control) mosquitoes was calculated through the method 2^−∆∆CT^ (Livak and Schmittgen, 2001) and statistically analyzed by the Student's t-test. Percent knockdown was calculated by subtracting the normalized 2^−∆∆CT^ (defined by the level of expression for dsGFP) and multiplying by 100.

**Figure S2. Box plot of data quality before and after preprocessing**. Representative images of quality values per base of the sample reads (A) before and (B) after initial quality processing with FastQC.

**Figure S3. Linear dynamic range and efficiency of the RT-qPCR assays**. Standard curves were generated by serially diluting cDNA. **A)** Primers for *lola* gene (Efficiency 110 %), **B)** Primers for Clip-domain gene (Efficiency 93.2 %), **C)** Primers for kayak gene (Efficiency 109.6 %), **D)** Primers for Draper gene (Efficiency 95.7 %), **E)** Primers for Cecropin gene (Efficiency 97.9 %), **F)** Primers for PPAF2 gene (Efficiency 99.6 %) and **G)** Primers for JNK gene (Efficiency 156.6 %). The logarithm to base 10 (log) of the cDNA copy number versus the cycle threshold (CT) value is represented. Each dot represents the Cq-value from two replicates, and the lines represent the best-fitting log-linear regression models.

**Figure S4. Overview of the functional categories of all differentially expressed genes (DEGs) in *lola*-silenced mosquitoes.** (A) GO enrichment analysis: GO terms from the biological functions and processes categories for differentially abundant 192 transcripts that were not induced by immune challenge in *lola*-silenced mosquitoes. The circle size indicates the number of genes included in the category. (B) Network visualization. Graph obtained with ShinyGO.

**Figure S5. Genes for components of the Toll and Imd signaling pathways that are differentially expressed in *lola* KD mosquitoes**. The components of this signaling pathway that were identified as differentially expressed in our analysis are highlighted in red. Pathway painting was performed with log2-fold change and the KEGG ID for each gene using SRplot. This color-coded map of DEGs corresponds to aali04624 in the KEGG database, corresponding to the signaling pathway model for *Anopheles albimanus.* The JNK pathway branches out from the Imd pathway.

**Figure S6. Genes for components of the lysosomal signaling pathway that are differentially expressed in *lola* KD mosquitoes**. The components of this signaling pathway that were identified as differentially expressed in our analysis are highlighted in red. Pathway painting was performed with log2-fold change and KEGG ID for each gene using SRplot. This color-coded map of DEGs corresponds to aali04142 in the KEGG database, corresponding to the signaling pathway model for *Anopheles albimanus.*

**Figure S7. Genes for components of the efferocytosis signaling pathway that are differentially expressed in *lola* KD mosquitoes.** The components of this signaling pathway that were identified as differentially expressed in our analysis are highlighted in red. Pathway painting was performed with log2-fold change and KEGG ID for each gene using SRplot. This color-coded map of DEGs corresponds to aali04148 in the KEGG database, corresponding to the signaling pathway model for *Anopheles albimanus.*

**References**

Livak KJ & Schmittgen TD. (2001). Analysis of relative gene expression data using real-time quantitative PCR and the 2(-Delta Delta C(T)) Method. Methods. Dec;25(4):402-8. doi: 10.1006/meth.
